# Genome-wide identification, characterization and expression analysis of AGO, DCL, and RDR families in *Chenopodium quinoa*

**DOI:** 10.1038/s41598-023-30827-1

**Published:** 2023-03-04

**Authors:** Shiyu Yun, Xin Zhang

**Affiliations:** 1 Institute of Industrial Crops, Shanxi Agricultural University, Taiyuan, 030031 China; 2 Present Address: State Key Laboratory of Sustainable Dryland Agriculture, Shanxi Agricultural University, Taiyuan, 030031 China

**Keywords:** Computational biology and bioinformatics, Physiology, Plant sciences

## Abstract

RNA interference is a highly conserved mechanism wherein several types of non-coding small RNAs regulate gene expression at the transcriptional or post-transcriptional level, modulating plant growth, development, antiviral defence, and stress responses. Argonaute (AGO), DCL (Dicer-like), and RNA-dependent RNA polymerase (RDR) are key proteins in this process. Here, these three protein families were identified in *Chenopodium quinoa*. Further, their phylogenetic relationships with Arabidopsis, their domains, three-dimensional structure modelling, subcellular localization, and functional annotation and expression were analysed. Whole-genome sequence analysis predicted 21 *CqAGO*, eight *CqDCL*, and 11 *CqRDR* genes in quinoa. All three protein families clustered into phylogenetic clades corresponding to those of Arabidopsis, including three AGO clades, four DCL clades, and four RDR clades, suggesting evolutionary conservation. Domain and protein structure analyses of the three gene families showed almost complete homogeneity among members of the same group. Gene ontology annotation revealed that the predicted gene families might be directly involved in RNAi and other important pathways. Largely, these gene families showed significant tissue-specific expression patterns, RNA-sequencing (RNA-seq) data revealed that 20 *CqAGO*, seven *CqDCL*, and ten *CqRDR* genes tended to have preferential expression in inflorescences. Most of them being downregulated in response to drought, cold, salt and low phosphate stress. To our knowledge, this is the first study to elucidate these key protein families involved in the RNAi pathway in quinoa, which are significant for understanding the mechanisms underlying stress responses in this plant.

## Introduction

RNA interference (RNAi), also known as RNA silencing, is an extremely important and highly conserved gene-expression regulatory mechanism widely distributed among eukaryotes. RNAi is mediated by small non-coding RNAs that regulate gene expression at the transcriptional and post-transcriptional levels by specifically identifying complementary RNA targets, and protecting cells against harmful exogenous and endogenous genetic elements^1,2^. Thus, RNAi plays an important role in the regulation of plant development, epigenetic modification, genome stability maintenance, and abiotic and biotic stress responses^3–5^. Argonaute (AGO), Dicer-like (DCL), and RNA-dependent RNA polymerase (RDR) are key proteins of the RNAi pathway^6^.

RNAi is initially triggered by double-stranded RNA (dsRNA) or partially double-stranded stem–loop RNA that is cleaved by DCL into 21–24-nt small RNA (sRNA) duplexes^7^, which are then incorporated with AGO protein to form the pre-RNA-induced silencing complex (pre-RISC) that requires the molecular chaperone Heat shock protein 70 (Hsp70)/90 (Hsp90)^8^. The duplex is melted by the action of the N-domain of AGO and only the guide RNA strand remains in the complex to form a mature RISC^9,10^. RISC binds to complementary mRNA guided by single-stranded sRNA to inhibit translation during post-transcriptional gene silencing (PTGS) or mediates DNA methylation and heterochromatin formation during transcriptional gene silencing (TGS), resulting in specific gene silencing^11,12^. RDR recognizes aberrant RNA and catalysing phosphodiester bond formation between ribonucleotides to synthesize other dsRNA, providing a new substrate to DCL, which can enhance RNAi signals or initiate a new round of RNAi^13^.

To date, several studies have shown that the sizes of *AGO*, *DCL*, and *RDR* gene families vary among species. For example, Arabidopsis^14^, rice^15^, maize^16^, millet^17^, grapevine^18^, tomato^19^, wheat^20^, soybean^21^, pepper^22^, cucumber^23^, barley^24^, sugarcane^25^, sweet orange^6^, and tea^26^ genomes encode ten, 19, 18, 19, 13, 15, 39, 21, 12, seven, 11, 21, eight, and 18 *AGO* genes; four, five, five, eight, four, seven, seven, seven, four, five, five, four, four, and five *DCL* genes; six, eight, five, 11, five, six, 16, seven, six, eight, seven, 11, four, and nine *RDR* genes, respectively. These studies have also shown that these gene families are highly conserved in plants, although little is known about the corresponding genes in quinoa.

Quinoa is a tetraploid dicotyledonous species with a cultivation history of over 7000 years. Its seeds can be consumed as entire grains or ground into flour, and its leaves and stems can be used as animal feed^27^. Quinoa is high in nutritional value and contains a variety of essential amino acids, fats, dietary fibre, vitamins, and minerals, among other valuable nutrients^28^. In addition, quinoa contains a large number of secondary metabolites, such as steroids, flavonoids, and triterpene saponins^29^, which have anti-microbial^27^, anti-diabetic^30^, anti-inflammatory^31^, and immunomodulatory activities^32^. Moreover, quinoa is resistant to salinity, frost, and drought, and can be planted in marginal environments. Therefore, quinoa has garnered increasingly widespread attention, and the year 2013 was declared ‘The International Year of Quinoa’ by the United Nations^33^.

Although quinoa exhibits excellent resistance to stress, the mechanisms at play are not well understood. Studies have shown that when plants are subjected to biotic or abiotic stress, the sRNAs involved in the RNAi pathway play an important role in the regulation of gene expression^34,35^. Here, we systematically studied the *AGO*, *DCL*, and *RDR* gene families in quinoa through whole genome analysis. We identified the evolutionary relationship of these gene families with those of Arabidopsis, and analysed the secondary domains, three-dimensional (3D) structure, subcellular localization, and functional annotation of the identified *AGO*, *DCL*, and *RDR* genes. The results reported herein provide further insights into the molecular mechanism of RNAi and will help understand the mechanisms underlying stress resistance in quinoa.

## Results

### Screening of *AGO*, *DCL*, and *RDR* genes in quinoa

To identify quinoa *AGO*, *DCL*, and *RDR* genes, the *Chenopodium quinoa* v1.0 database was searched for the transcripts of each gene family that contained the characteristic domains. The assigned names, primary transcript ID, chromosome localization, description of main transcripts, CDS, and peptide lengths are shown in Tables 1 and 2. A total of 25 *AGO*, 12 *DCL,* and 12 *RDR* genes were initially recognized, and after considering the lack or overlap of the functional domain and insufficient length of the amino acid (aa) sequence, 21 *AGO*, eight *DCL*, and 11 *RDR* genes were ultimately identified. The gene IDs of *AtAGOs*, *AtDCLs*, and *AtRDRs* are shown in Table S1.

**Table 1:** Information about the predicted *CqAGO* gene families.

Assigned name	Primary transcript ID (phytozome)	Locus	Description	CDS length (bp)	Peptide length (aa)
*CqAGO1a*	AUR62038571 (PAC:36304424)	[Chr01]: 1324761 … 1333707	Similar to AGO1 protein argonaute 1 (*Arabidopsis thaliana*)	3165	1055
*CqAGO1b*	AUR62042041 (PAC:36293791)	[Chr02]: 16801641 … 16810449	Similar to AGO1 protein argonaute 1 (*Arabidopsis thaliana*)	3048	1016
*CqAGO2/3*	AUR62042863 (PAC:36297668)	[Chr14]: 46903941 … 46907092	Similar to AGO2 protein argonaute 2 (*Arabidopsis thaliana*)	1953	651
*CqAGO4a*	AUR62020961 (PAC:36282815)	[Chr11]: 1919738 … 1924859	Similar to AGO7 protein argonaute 7 (*Oryza sativa* subsp. japonica)	2529	843
*CqAGO4b*	AUR62018467 (PAC:36291322)	[Chr07]: 86000200 … 86005236	Similar to AGO8 protein argonaute 8 (*Arabidopsis thaliana*)	2547	819
*CqAGO4c*	AUR62020956 (PAC:36282898)	[Chr11]: 1870101 … 1886659	Similar to AGO4 protein argonaute 4 (*Arabidopsis thaliana*)	2610	870
*CqAGO4d*	AUR62020957 (PAC:36282782)	[Chr11]: 1890564 … 1895492	Similar to iwi Piwi-like protein (*Dugesia japonica*)	2328	776
*CqAGO4e*	AUR62018466 (PAC:36291279)	[Chr07]: 86008990 .. 86013956	Similar to AGO4 protein argonaute 4 (*Arabidopsis thaliana*)	2598	866
*CqAGO4f*	AUR62018462 (PAC:36291336)	[Chr07]: 86140248 … 86145122	Similar to AGO4 protein argonaute 4 (*Arabidopsis thaliana*)	2841	827
*CqAGO5a*	AUR62023979 (PAC:36289833)	[Chr15]: 24464764 … 24471406	Similar to AGO5 protein argonaute 5 (*Arabidopsis thaliana*)	2601	867
*CqAGO5b*	AUR62031777 (PAC:36288849)	[Chr18]: 13090421 … 13096877	Similar to MEL1 protein argonaute MEL1 (*Oryza sativa* subsp. japonica)	2610	870
*CqAGO5c*	AUR62031774 (PAC:36288928)	[Chr18]: 13005734 … 13011950	Similar to AGO5 protein argonaute 5 (*Arabidopsis thaliana*)	2802	934
*CqAGO5d*	AUR62023977 (PAC:36289945)	[Chr15]: 24386478 … 24394217	Similar to MEL1 protein argonaute MEL1 (*Oryza sativa* subsp. *japonica*)	2814	938
*CqAGO5e*	AUR62031775 (PAC:36288861)	[Chr18]: 13053350 … 13060228	Protein of unknown function	2253	751
*CqAGO6a*	AUR62017582 (PAC:36291384)	[Chr00]: 136398546 … 136407125	Similar to AGO16 protein argonaute 16 (*Oryza sativa* subsp. *japonica*)	2535	845
*CqAGO6b*	AUR62015121 (PAC:36318843)	[Chr15]: 58303924 … 58311626	Similar to AGO16 protein argonaute 16 (*Oryza sativa* subsp. *japonica*)	2703	901
*CqAGO7a*	AUR62032845 (PAC:36324041)	[Chr06]: 6044761 … 6048102	Similar to AGO7 protein argonaute 7 (*Arabidopsis thaliana*)	2841	947
*CqAGO7b*	AUR62005852 (PAC:36320054)	[Chr14]: 58204384 … 58207699	Similar to AGO7 protein argonaute 7 (*Arabidopsis thaliana*)	2844	948
*CqAGO8/9*	AUR62037160 (PAC:36299083)	[Chr18]: 11113121 … 11123878	Similar to AGO4B protein argonaute 4B (*Oryza sativa* subsp. *japonica*)	2928	976
*CqAGO10a*	AUR62033429 (PAC:36284202)	[Chr01]: 19947679 … 19953895	Similar to PHN1 protein argonaute PNH1 (*Oryza sativa* subsp. *japonica*)	2823	941
*CqAGO10b*	AUR62011053 (PAC:36312551)	[Chr02]: 56071707 … 56077939	Similar to PHN1 protein argonaute PNH1 (*Oryza sativa* subsp. *japonica*)	2823	941

**Table 2:** Information about the predicted *CqDCL* and *CqRDR* gene families.

Assigned name	Primary transcript ID (phyzome)	Locus	Description	CDS length (bp)	Peptide length (aa)
*CqDCL*					
* CqDCL1a*	AUR62004575 (PAC:36318980)	[Chr01]: 120585852 … 120595683	Similar to DCL1 endoribonuclease dicer homolog 1 (*Arabidopsis thaliana*)	5637	1879
* CqDCL1b*	AUR62022688 (PAC:36312037)	[Chr10]: 5693359 … 5703084	Similar to DCL1 endoribonuclease dicer homolog 1 (*Arabidopsis thaliana*)	5658	1886
* CqDCL2a*	AUR62006631 (PAC:36302641)	[Chr05]: 77485434 … 77494612	Similar to At3g03300 endoribonuclease dicer homolog 2 (*Arabidopsis thaliana*)	4083	1361
* CqDCL2b*	AUR62000283 (PAC:36298082)	[Chr12]: 3351865 … 3361389	Similar to At3g03300 endoribonuclease dicer homolog 2 (*Arabidopsis thaliana*)	3990	1330
* CqDCL3a*	AUR62036713 (PAC:36294753)	[Chr03]: 5531087 … 5547047	Similar to DCL3 endoribonuclease dicer homolog 3 (*Arabidopsis thaliana*)	4839	1613
* CqDCL3b*	AUR62013883 (PAC:36307361)	[Chr10]: 15653223 … 15656198	Similar to DCL3 endoribonuclease dicer homolog 3 (*Arabidopsis thaliana*)	2976	992
* CqDCL4a*	AUR62042026 (PAC:36306186)	[Chr01]: 32971126 … 32993157	Similar to DCL4 dicer-like protein 4 (*Arabidopsis thaliana*)	7065	2355
* CqDCL4b*	AUR62011271 (PAC:36312559)	[Chr02]: 59016581 … 59032884	Similar to DCL4 dicer-like protein 4 (*Arabidopsis thaliana*)	4899	1633
*CqRDR*					
* CqRDR1*	AUR62030555 (PAC:36281681)	[Chr04]: 48216145 … 48221258	Similar to RDR1 RNA-dependent RNA polymerase 1 (*Arabidopsis thaliana*)	3369	1123
* CqRDR2a*	AUR62030995 (PAC:36316990)	[Chr08]: 6725418 … 6731476	Similar to RDR2 RNA-dependent RNA polymerase 2 (*Arabidopsis thaliana*)	3213	1071
* CqRDR2b*	AUR62008243 (PAC:36286666)	[Chr16]: 2103802 … 2106913	Similar to RDR2 RNA-dependent RNA polymerase 2 (*Arabidopsis thaliana*)	2220	740
* CqRDR3a*	AUR62021424 (PAC:36313669)	[Chr01]: 17717880 … 17729029	Similar to RDR1 probable RNA-dependent RNA polymerase 1 (*Oryza sativa* subsp. *japonica*)	2556	852
* CqRDR3b*	AUR62010903 (PAC:36312680)	[Chr02]: 54077402 … 54089040	Similar to RDR3 probable RNA-dependent RNA polymerase 3 (*Arabidopsis thaliana*)	3066	1022
* CqRDR3c*	AUR62042309 (PAC:36300346)	[Chr18]: 936324 … 943470	Similar to RDR5 probable RNA-dependent RNA polymerase 5 (*Arabidopsis thaliana*)	1677	559
* CqRDR3d*	AUR62021425 (PAC:36313508)	[Chr01]: 17731358 … 17745690	Protein of unknown function	2574	858
* CqRDR3e*	AUR62010902 (PAC:36312633)	[Chr02]: 54059497 … 54071156	Similar to RDR1 RNA-dependent RNA polymerase 1 (*Arabidopsis thaliana*)	2643	881
* CqRDR3f*	AUR62021426 (PAC:36313611)	[Chr01]: 17754867 … 17766351	Similar to RDR5 probable RNA-dependent RNA polymerase 5 (*Arabidopsis thaliana*)	2850	950
* CqRDR6a*	AUR62012402 (PAC:36280582)	[Chr03]: 76755341 … 76759523	Similar to RDR6 RNA-dependent RNA polymerase 6 (*Arabidopsis thaliana*)	3561	1187
* CqRDR6b*	AUR62031504 (PAC:36317221)	[Chr04]: 230722 … 234878	Similar to RDR6 RNA-dependent RNA polymerase 6 (*Arabidopsis thaliana*)	3624	1208

**Figure 1:**
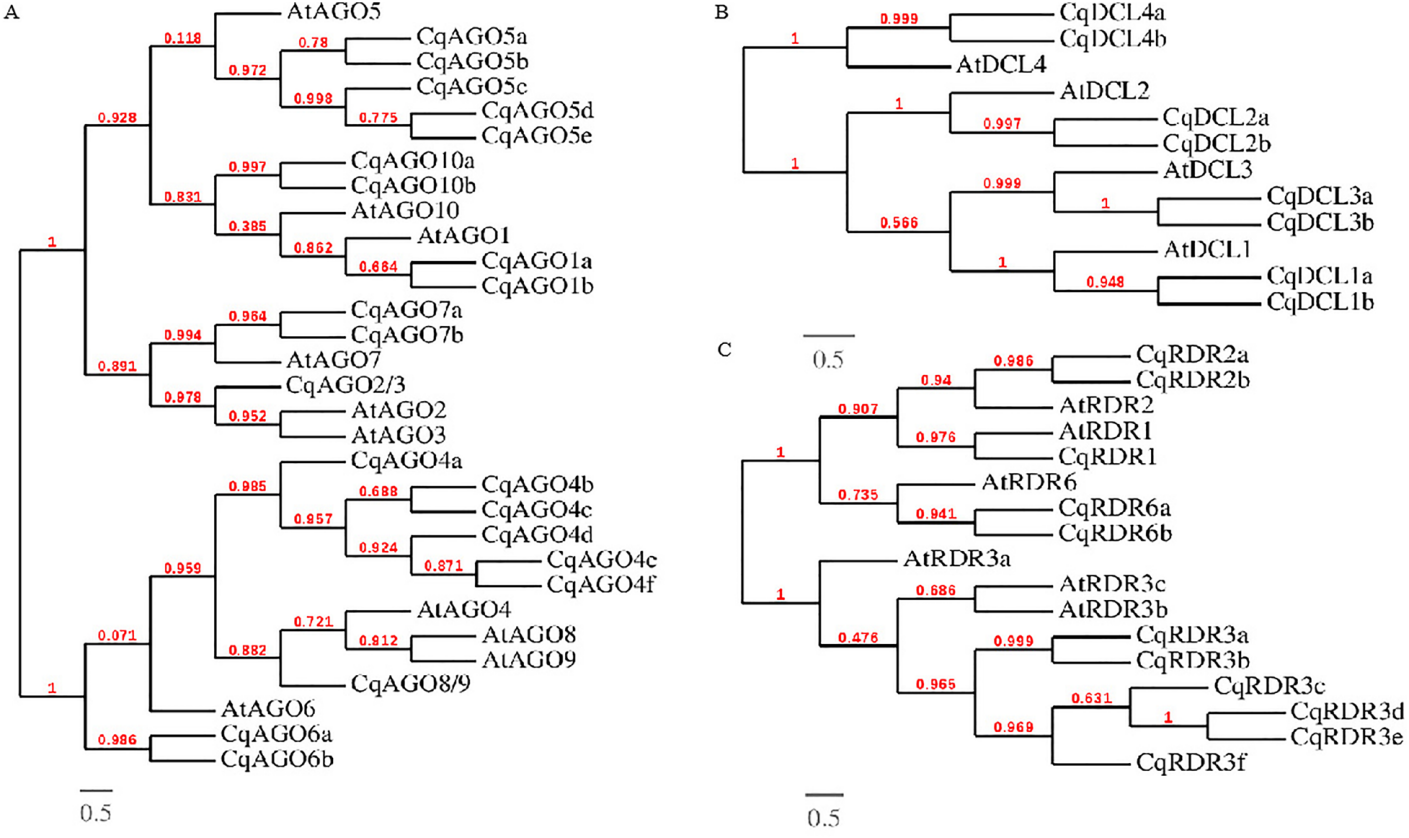
Phylogenetic analysis. (**A**) Relationship between AtAGO and CqAGO proteins. (**B**) Relationship between AtDCL and CqDCL proteins. (**C**) Relationship between AtRDR and CqRDR proteins. Branch length was ignored, and branch support values are displayed. The scale bar at the bottom left corner represents the branch length.

**Figure 2:**
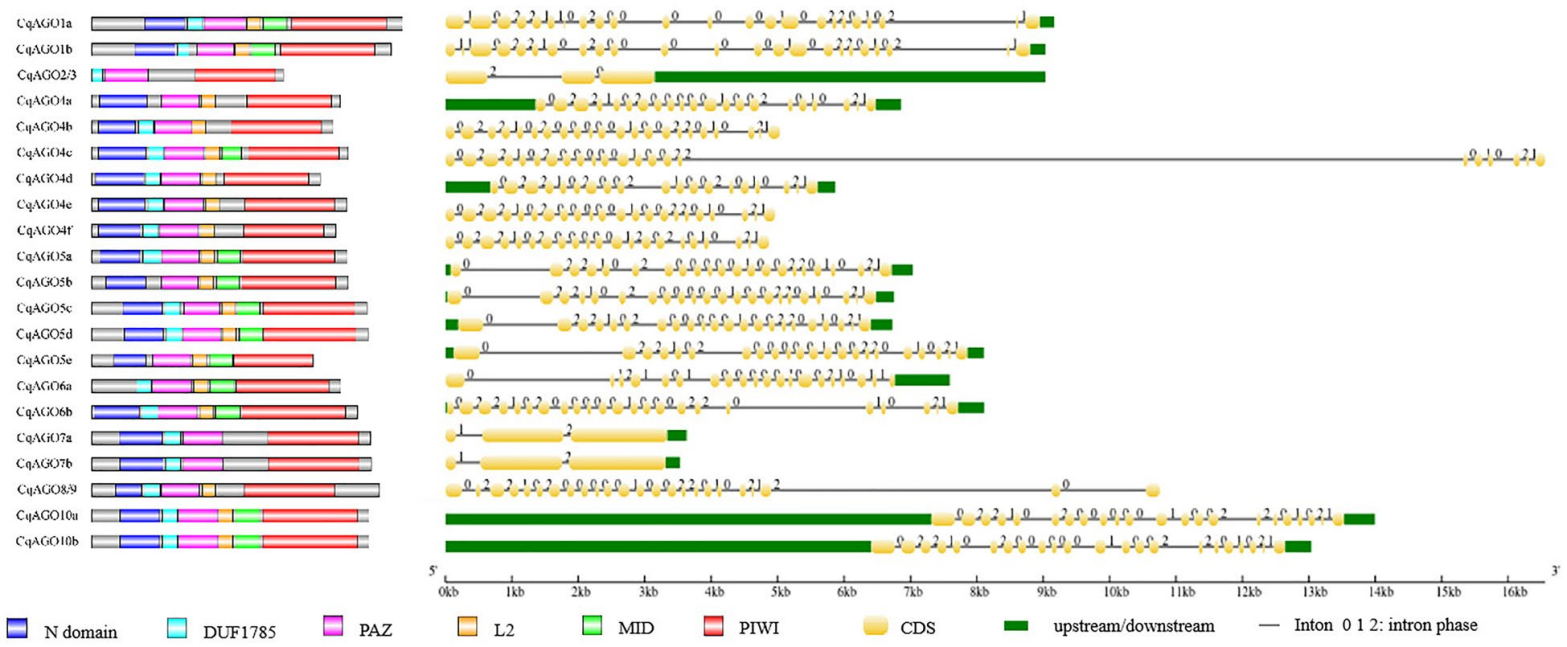
Conserved domains of CqAGO proteins identified by SMART and Pfam, generated using IBS (left). The protein domains include N domain, DUF1785, PAZ, L2, MID, and PIWI. Introns in *CqAGO* genes are shown on the right.

**Figure 3:**
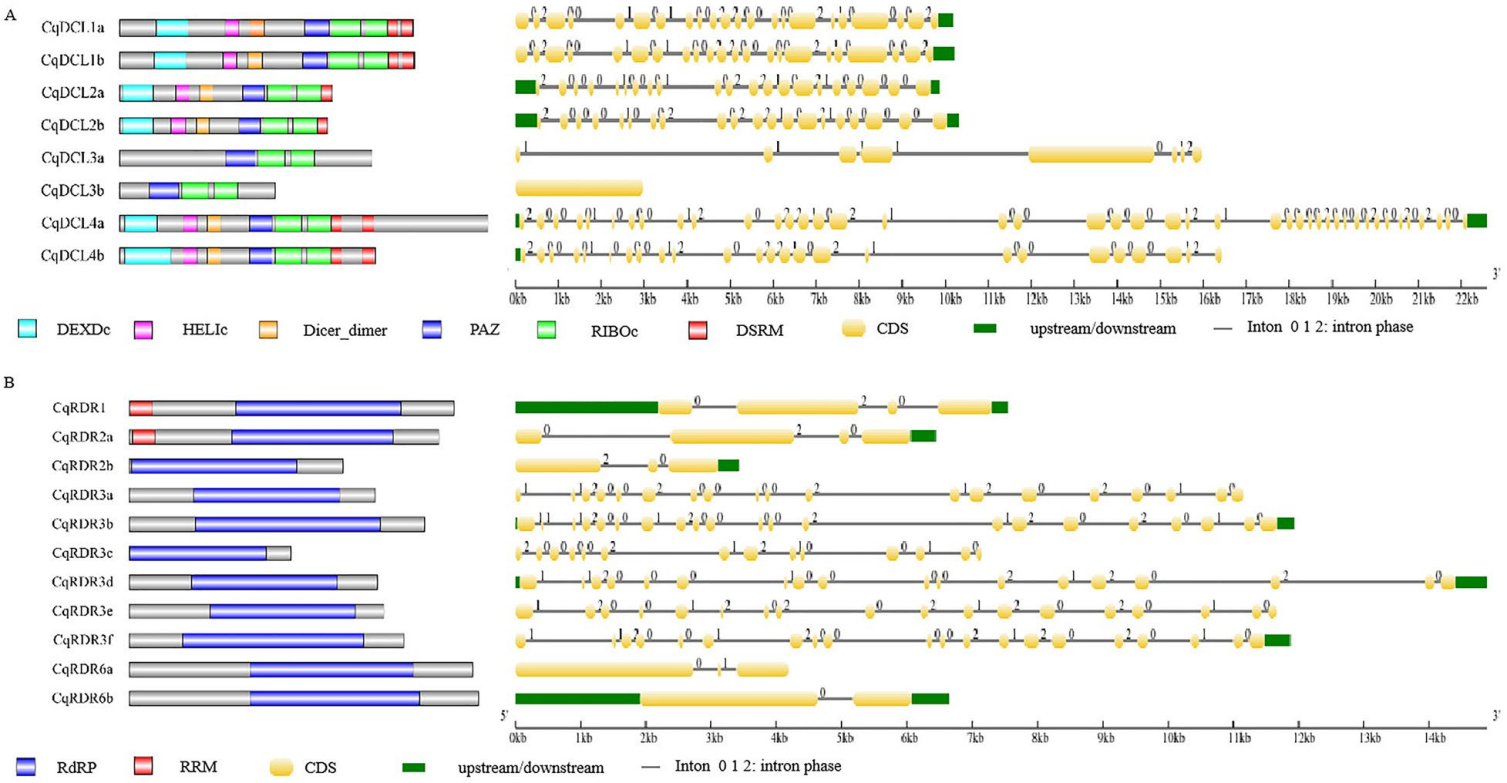
Domain structure of CqDCLs and CqRDRs (left). (**A**) Conserved domains of CqDCL proteins identified by SMART and Pfam, and generated using IBS. The protein domains include DEXDc, HELICc, Dicer-dimer, PAZ, RIBOc, and DSRM. (**B**) Conserved domains of CqRDR proteins identified by SMART and Pfam, and generated by IBS. The protein domains include RRM and RdRP. Introns in *CqDCL* and *CqRDR* genes are shown on the right.

A total of 21 *CqAGO* homologues were localized on nine chromosomes and mostly concentrated on chromosomes 07, 11, 15, and 18. Chromosomes 07, 11, and 15 harbor three *CqAGO* genes, and chromosome 18 harbors four *CqAGO* genes (Fig. S1). The length of the CDS ranged from 1953 to 3165 bp (Table 1). Most *CqAGO* genes possessed 18–23 introns, whereas *CqAGO2/3*, *CqAGO7a*, and *CqAGO7b* contained two introns each, where they were localized in the AtAGO2/3/7 clade (Figs. 1A and 2). Two *CqDCL* genes were detected on chromosomes 01 and 10, one *CqDCL* gene was localized to chromosomes 02, 03, 05, and 12 (Fig. S1). CDS length varied from 2976 to 7065 bp, produced by *CqDCL10b* and *CqDCL1a*, with coding proteins of 992 and 2355 aa, respectively. The number of introns varied from 0 to 42 in *CqDCLs* (Fig. 3A). *CqRDR* genes were mainly present on chromosomes 01, 02, and 04 (Fig. S1), and the length of the CDS ranged from 1677 to 3624 bp (Table 2). There were significant differences in the number of introns among *CqRDR* members, and the intron numbers of *CqRDR1*, *2a*, 2*b*, *6a*, and *6b*, which were localized in the AtRDR1/2/6 clade, were concentrated in the range from 1 to 3. In the AtRDR3 clade, *CqRDR3c* possessed only 13 introns, whereas the other *CqRDRs* contained a significantly higher number of introns, i.e., 17–20 (Figs. 1C and 3B).

### Phylogenetic tree and domain analysis of CqAGO, CqDCL, and CqRDR proteins

To determine the potential function of critical proteins in the RNAi pathway, we predicted the domains of 21 CqAGOs, eight CqDCLs, and 11 CqRDRs using SMART. Detailed prediction data, and corresponding confidence values of the domains of CqAGO (Table S2 and Fig. 2), CqDCL (Table S3 and Fig. 3A), and CqRDR (Table S4 and Fig. 3B) were obtained from SMART/Pfam, and visually analysed. Visual analysis of protein domains revealed similarities and differences in the position of typical conserved domains among members of each protein family. We found that CqAGO, CqDCL, and CqRDR had more copies than those of Arabidopsis, which further indicates that they may be functionally more diverse.

Phylogenetic analysis showed that the AGO protein sequences of Arabidopsis can be divided into three clades: AtAGO1/5/10, AtAGO2/3/7, and AtAGO4/6/8/9. We observed that CqAGO proteins were placed in all these clades; there were the following three CqAGOs: CqAGO2/3, 7a, and 7b within the AtAGO2/3/7 clade, CqAGO7a and CqAGO7b were grouped with the AtAGO7 clade, although they were localized on different chromosomes, and the sequence similarity between them was as high as 98.2% at the aa level. Furthermore, CqAGO7a and CqAGO7b shared 70% sequence similarity with AtAGO7. The AtAGO1/5/10 and AtAGO4/6/8/9 clades were highly diverse in quinoa, with nine CqAGO proteins. CqAGO1a, 1b, 5a, 5b, 5c, 5d, 5e, 10a, and 10b clustered into the AtAGO1/5/10 clade, whereas CqAGO4a, 4b, 4c, 4d, 4e, 4f, 6a, 6b, and 8/9 clustered into the AtAGO4/6/8/9 clade (Fig. 1A).

**Figure 4:**
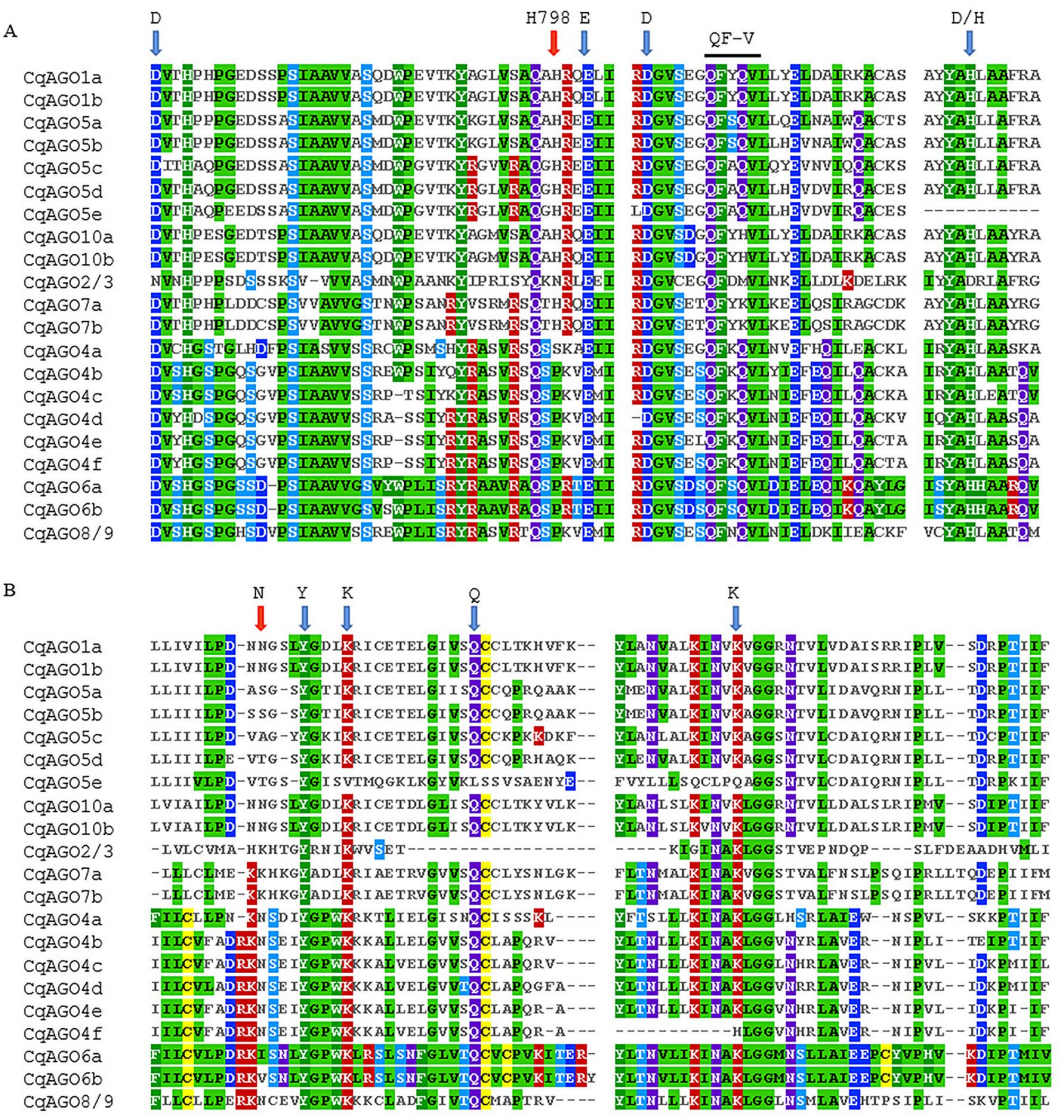
Functionally conserved amino acids of CqAGO proteins. (**A**) DEDD/H tetrad (blue arrows), H798 (red arrow) and QF-V motif within PIWI domains. (**B**) 5′-terminal nucleotide selection N (red arrow), 5′-phosphate-binding selection YKQK (blue arrows).

Consistent with other eukaryotic AGO proteins, four typical characteristic domains, including N domain, PIWI/Argonaute/Zwille (PAZ), middle (Mid), and p-body-induced wimpy tests (PIWI) domains, were found in several CqAGO proteins (Fig. 2), and the order of functional domains was consistent with AtAGO proteins. All CqAGO proteins had the PAZ and PIWI domains. Most predicted CqAGOs identified a variable N-t domain, which is composed of an N domain and a DUF1785 domain; CqAGO2/3 and CqAGO6a contained only the DUF1785 domain, whereas CqAGO4a, CqAGO5b and CqAGO5e contained only the N domain. In addition, all CqAGO proteins in the AtAGO2/3/7 clade did not contain the MID domain, whereas all CqAGOs in the AtAGO1/5/10 clade were predicted to contain the MID domain. AGOs in the same clade had high structural similarity, indicating that they may exhibit high functional similarity. MSA analysis of the PIWI domain of CqAGO proteins exhibited a conserved QF-V (Q = glutamine, F = phenylalanine, V = valine) motif and the metal-chelating residue motif DEDD/H (D = aspartic acid, E = glutamate, D = aspartic acid, and H = histidine) required for cleavage activity, except for CqAGO2/3 and CqAGO5e; the first D in CqAGO2/3 was replaced by N (asparagine), and CqAGO5e lacked the D/H residue of the catalytic tetrad (Fig. 4A). Moreover, Arabidopsis H798 (H798 of AtAGO1) is a very important aa residue, and most CqAGOs in the AtAGO1/5/10 and AtAGO2/3/7 clades retained H residues. H was replaced by N in CqAGO2/3. Furthermore, almost all H residues were replaced by P (proline) in the AtAGO4/6/8/9 clade, and was only replaced by S (serine) in CqAGO4a (Fig. 4A). Residues Y (tyrosine), K (lysine), Q, and K which are related to 5′- phosphate binding in sRNA, were completely conserved in all CqAGOs, except for CqAGO2/3, CqAGO4f, and CqAGO5e. Additionally, CqAGO2/3 lacked the conserved residue Q, while CqAGO5e only retained the residue Y, and the second K was replaced by H in CqAGO4f (Fig. 4B). The N residue, preferentially bound to 5′U 21-nt sRNA^36^, was conserved in CqAGO1a, 1b, 4a, 4b, 4c, 4d, 4e, 4f, 8/9, 10a, and 10b (Fig. 4B).

**Figure 5:**
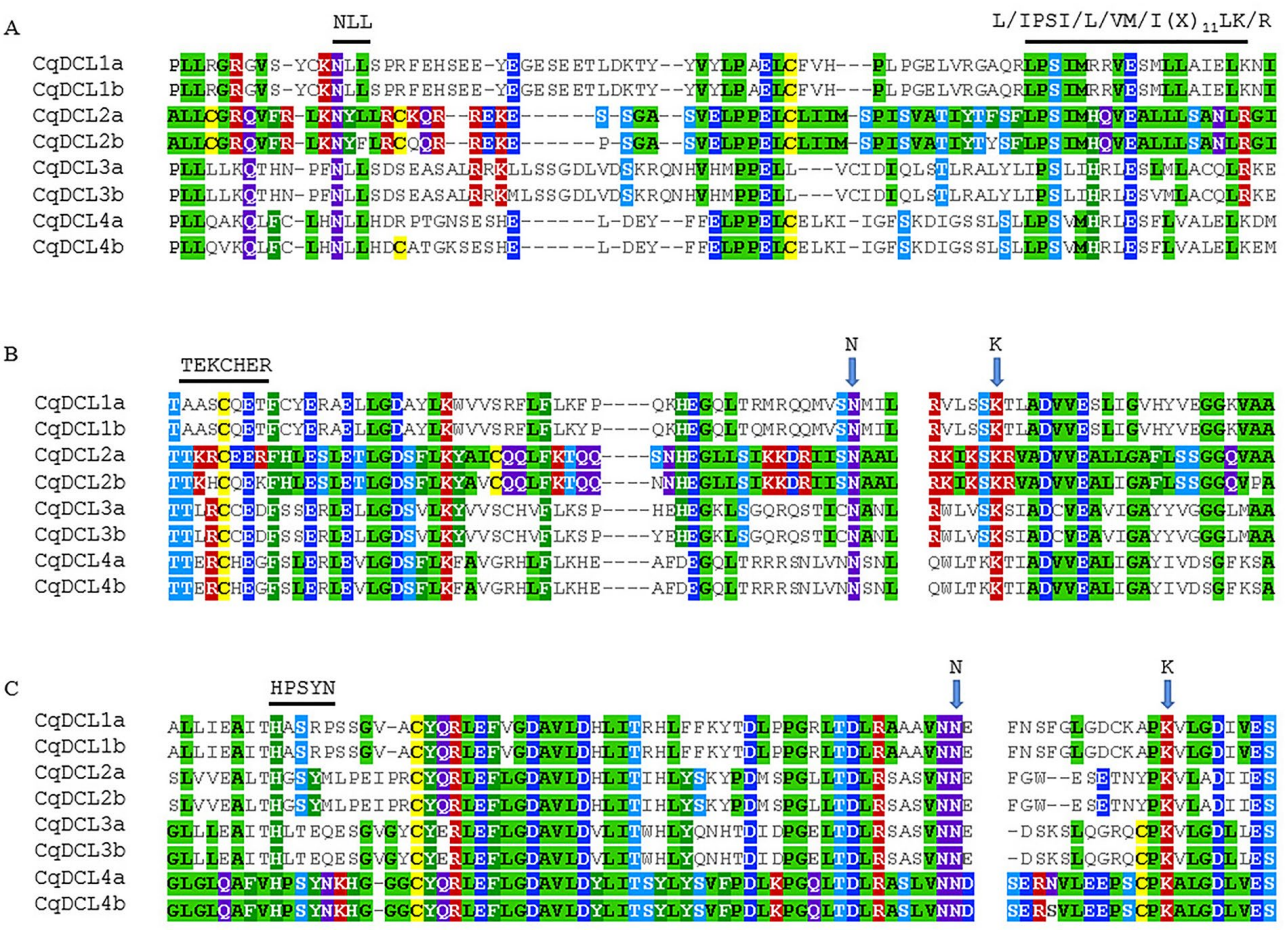
Functionally conserved amino acids in CqDCL proteins. (**A**) NLL motif and connector helix core L/IPSI/L/VM/I(X)11LK/R. (**B**) RNase III A TEKCHER motif, N, and K residues. (**C**) RNase III B HPSYN motif, N, and K residues.

Compared to Arabidopsis, the CqDCL families were more diverse, with the number of members exceeding those of Arabidopsis (four AtDCL members from four clades). Each member of the AtDCL expanded into two copies in quinoa (Fig. 1B). Analysis of the CqDCL proteins revealed that most CqDCL proteins consist of DEAD-like helicase superfamily (DEXDc), helicase superfamily C-terminal (HELICc), Dicer dimerization (Dicer-dimer or DUF283), PAZ, Ribonuclease III family (RIBOc), and double-stranded RNA-binding motif (DSRM) domains. CqDCL1a, CqDCL1b, CqDCL4a, and CqDCL4b contained all characteristic domains. CqDCL2a and CqDCL2b contained only one DSRM domain, whereas CqDCL3a and CqDCL3b contained only the PAZ and RIBOc domains (Fig. 3A). MSA analysis of CqDCL proteins showed that L/IPSI/L/VM/I(X)11LK/R in the core region of the connecting helix is relatively conserved. Except for CqDCL2a and CqDCL2b in the AtDCL2 clade, the NLL motif of the PAZ loop was responsible for connection with dsRNA in other CqDCLs were conserved (Fig. 5A). RIBOc has two domains: RNase IIIA and RNase IIIB. The TEKCHER motif of RNase IIIA and the HPSYN loop of RNase IIIB in AtDCL4 may interact with dsRNA^37^, but limited conservation was observed in quinoa; only the HPSYN loops in CqDCL4a and CqDCL4b were fully conserved. In addition, the catalytic aa residues N and K in the RIBOc domain were highly conserved (Fig. 5B,C).

**Figure 6:**
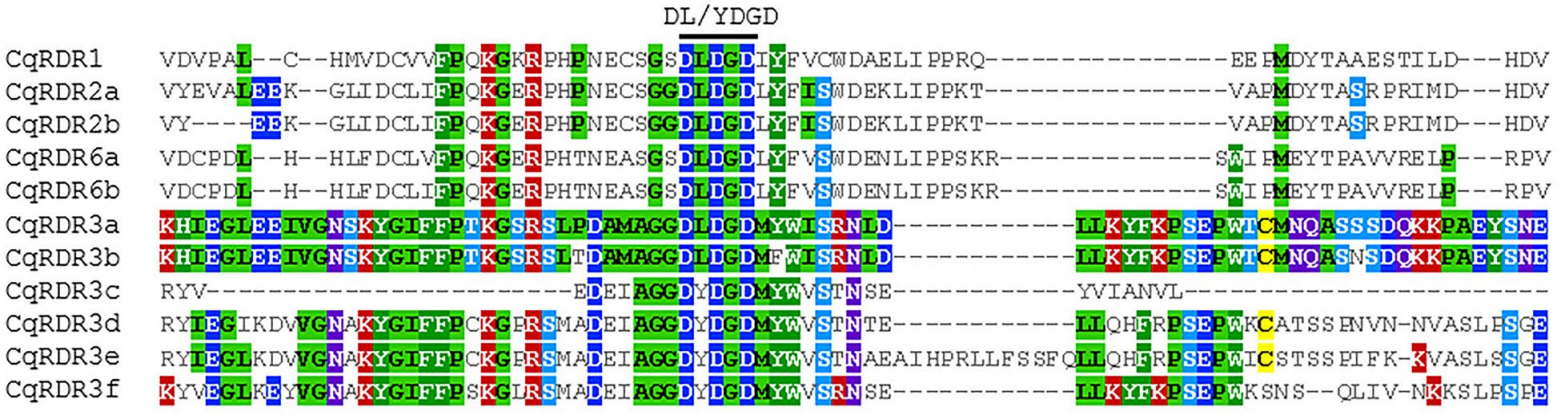
Functionally conserved DL/YDGD motif in CqRDR proteins.

According to phylogenetic analysis, AtRDR proteins were grouped into four clades: RDR1, RDR2, RDR3, and RDR6. There were six CqRDR proteins (CqRDR3a, 3b, 3c, 3d, 3e, and 3f) grouped together with RDR3, and the RDR1, RDR2, RDR6 clade contained one, two, two CqRDR proteins, respectively (Fig. 1C). CqRDR6a and CqRDR6b, belonging to the AtRDR6 clade, shared 96% sequence similarity. Structural analysis of the CqRDR proteins showed that the RNA-dependent RNA polymerase (RdRP) domain was present in all predicted CqRDRs; CqRDR1 and CqRDR2a had an RNA-recognition motif (RRM) domain (Fig. 3B). Moreover, CqRDR1, 2a, 2b, 6a, 6b, 3a, and 3b possessed canonical DLDGD, whereas CqRDR3c, CqRDR3d, CqRDR3e, and CqRDR3f contained DYDGD (Fig. 6).

### 3D modelling of CqAGO proteins

SWISS-MODEL is the best software for protein 3D model prediction^38^. We used SWISS-MODEL to obtain a 3D model of CqAGO and verified the predicted structure using four different measures.

**Figure 7:**
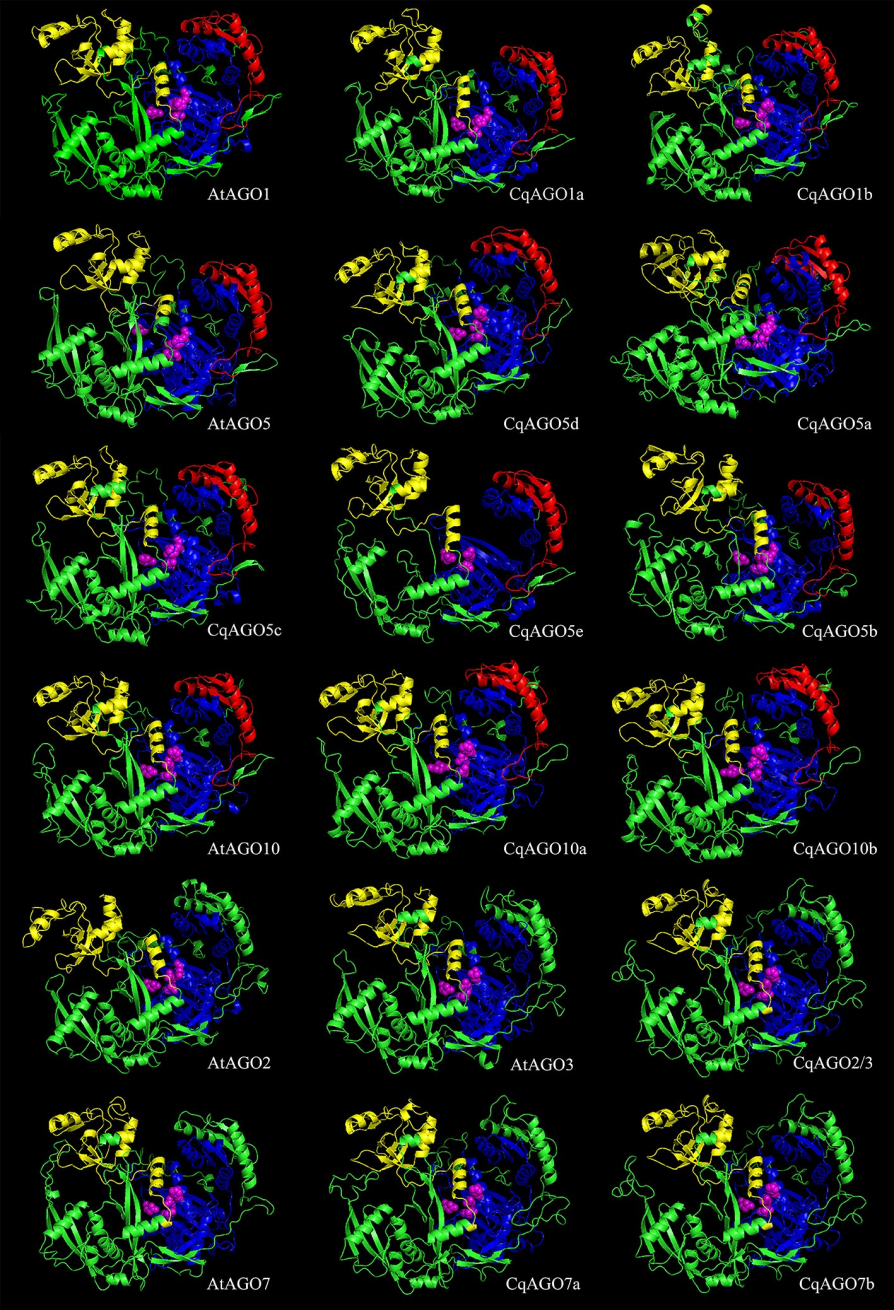
3D structure predictions for the AtAGO1/5/10 and AtAGO2/3/7 clades, as predicted using SWISS-MODEL. PAZ (yellow), PIWI (blue), and MID (red) domains as predicted using SMART and Pfam are displayed. DEDD/H is marked by magenta spheres.

QMEAN and GMQE are two different measures for evaluating models in SWISS-MODEL. QMEAN is based on a single model that is used to derive the absolute quality mass of each residue and the entire structure. The QMEAN *z*-score of a high-quality model should be between − 4.0 and 0. In turn, GMQE combines the attributes of target-template alignment and template structure, with values between 0 and 1. The larger the score, the more reliable the quality of the predicted structure. This study also used PROCHECK, ERRAT, Verify 3D, and WHATCHECK to evaluate the quality of the model, with higher values indicating a better model in each case. The results are shown in Table S5. Figures 7 and S2 demonstrate the models of CqAGOs and the corresponding AtAGOs in the same clade. The predicted structure of CqAGOs was similar to that of the corresponding AtAGOs, suggesting a high degree of functional conservation.

### Function prediction and subcellular localization

To better understand the biological functions of *CqDCL*, *CqAGO*, and *CqRDR*, Expasy was used to perform gene ontology (GO) annotations. The GO annotations for *CqDCL* and *CqRDR* were relatively complete. In the *CqAGO* family, *CqAGO5a* had the most comprehensive annotations, it participates in miRNA binding (GO:0035198), miRNA loading onto RISC involved in gene silencing by miRNA (GO:0035280), and miRNA-mediated inhibition of translation (GO:0035278), all of which are closely related to the RNAi pathway. *CqAGO1a* is involved in miRNA binding (GO:0035198) and gene silencing by miRNA (GO:0035195). Eight *CqRDR* genes play a role in the production of small interfering RNA (siRNA) involved in chromatin silencing by small RNA (GO:0070919) and the production of siRNA involved in RNA interference (GO:0030422). Moreover, 12 genes (eight *AGOs* and four *RDRs*) are involved in nucleic acid binding (GO:0003676), four genes (three *DCLs* and one *RDR*) are involved in RNA binding (GO:0003723), and *CqAGO7a* is involved in RNA interference (GO:0016246) (Tables S6–8). Most *CqAGOs*, *CqDCLs*, and *CqRDRs* showed some annotations related to RNAi, indicating that these genes are closely related to the RNAi pathway in quinoa.

Most CqAGOs are located in the nucleus, except for CqAGO4a (predicted to localize in the cytosol), CqAGO5a (predicted to localize in the mitochondrion and chloroplast), and CqAGO6a and CqAGO6b (predicted to localize in the cytosol and mitochondrion). All CqDCLs are localized in the nucleus, CqDCL1a, CqDCL1b, CqDCL3a, and CqDCL4b are also localized in the cytosol, and CqDCL2b is also localized in the membrane. As for CqRDRs, CqRDR3a, CqRDR3c, CqRDR3f, and CqRDR6b are localized in the cytosol and chloroplast, CqRDR2a, CqRDR3d, and CqRDR 3e are localized only in the cytosol, CqRDR1 and CqRDR3b are localized only in the nucleus, CqRDR6a is localized in the nucleus and cytosol, and CqRDR2b is localized in the nucleus and mitochondrion (Table S9).

### Expression profiles of *CqAGOs*, *CqDCLs*, and *CqRDRs*

**Figure 8:**
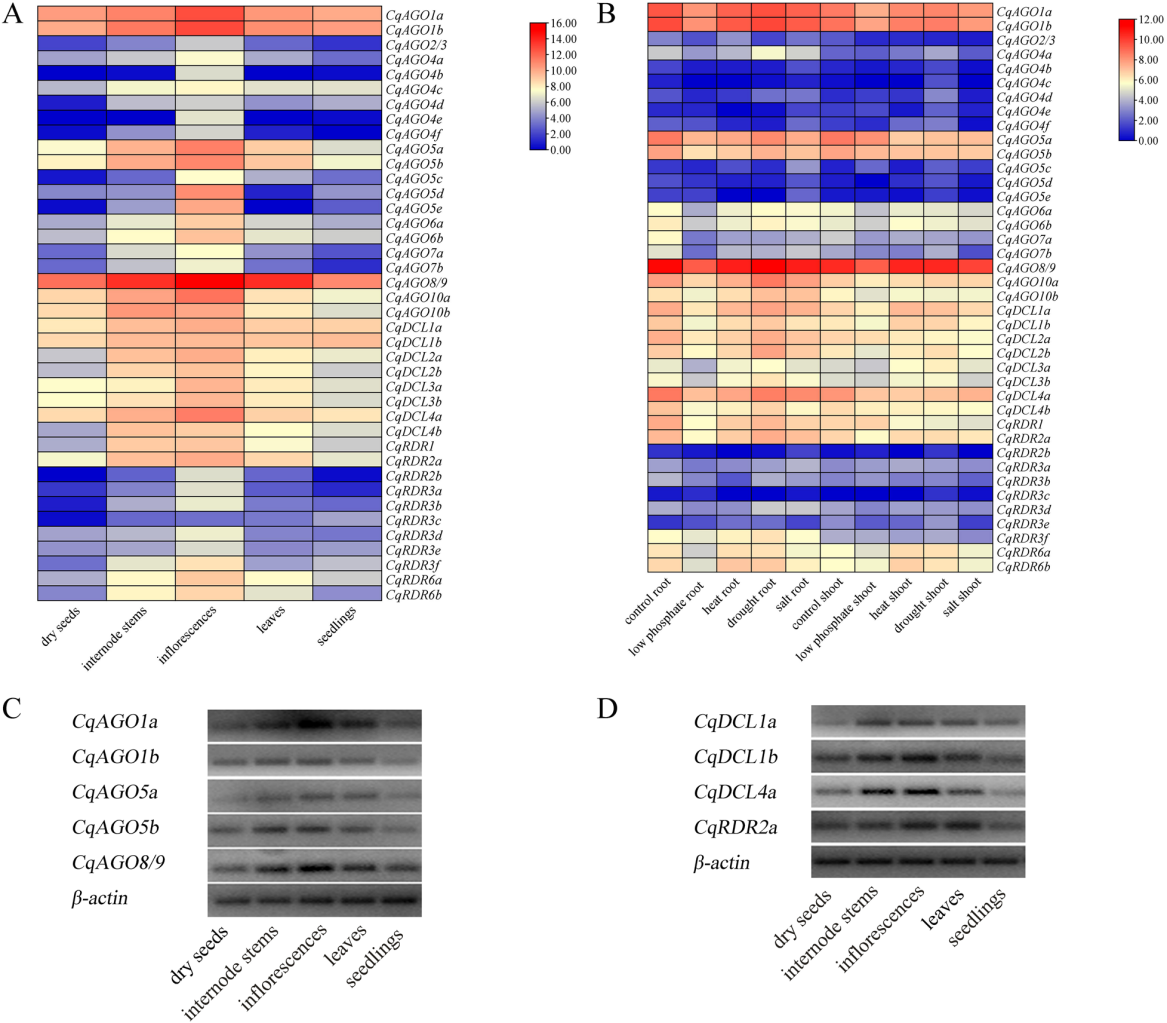
(**A**) Expression profiles of *CqAGO*s, *CqDCL*s, and *CqRDR*s in different tissues, including dry seeds; one-week-old seedlings; stems, leaves, and inflorescences of six-week-old plants. (**B**) Expression profiles of *CqAGO*s, *CqDCL*s, and *CqRDR*s under low-phosphate, heat, drought, and salt stresses in root and shoot, respectively. The log2 normalized value of original TPM data are represented in both figures. The colour bar at the right of the heat map represents relative expression values. Electrophoresis analysis in different tissues of *CqAGOs* (**C**) and *CqDCLs*, *CqRDR* (**D**). *β-actin* was used as a control for each tissue types.

The RNA-seq results showed that eight *CqDCLs* were expressed in dry seeds, one-week-old seedlings, stems, leaves, and inflorescences from six-week-old plants. *CqAGO4b*, *CqAGO4e,* and *CqRDR2b* were not expressed in dry seeds, while *CqAGO4b*, and *CqAGO5e* were not detected in leaves. Most of the *CqAGOs*, *CqDCLs* and *CqRDRs* had the highest expression levels in inflorescences. Only *CqAGO10b* and *CqDCL4b* had the highest expression in internode stems, and *CqRDR3c* had the highest expression in seedlings (Fig. 8A). In addition, most *CqAGO*, *CqDCL* and *CqRDR* genes responded to drought, heat, salt and low phosphorus stresses, being up- or downregulated. The expression levels of *CqAGO5a*, *CqAGO5b*, *CqAGO7a*, *CqDCL2a*, *CqDCL4a*, and *CqDCL4b* all showed a downward trend under the four stresses in the two tissues (Fig. 8B). According to the result of RNA-seq, 11 candidate genes were screened. Because of the high homology of *CqAGO10a* and *CqAGO10b*, we could not design primers with high specificity. Thus, five, three, and one genes from *CqAGOs*, *CqDCL*s, and *CqRDRs*, respectively, were chosen to analysed by electrophoresis. The electrophoresis results showed that all the tested genes were expressed in five tissues, with high expression in the stems, leaves, and inflorescences, and relatively low expression in seedlings and dry seeds (Fig. 8C,D). These findings were consistent with the RNA-seq results.

## Discussion

In this study, we investigated the phylogenetic relationship, structure, and functions of CqAGO, CqDCL, and CqRDR proteins. Our results showed that, compared to Arabidopsis, CqAGO, CqDCL, and CqRDR exhibited more copies and may thus have higher functional diversification. We discuss the phylogenetic relationships and predict structural domains in detail.

The AGO protein is the main executive element of RISC and the main effector of RNAi. In Arabidopsis*,* AtAGO4, 6, and 9 in the AGO4/6/8/9 clade are mainly bound to 24-nt siRNA and are responsible for RNA-directed DNA methylation^39,40^. In turn, AGO proteins in the AGO1/5/10 clade participate in the regulation of plant development and stress responses. For example, AGO1 binds to miR156 in seedlings, is involved in shoot development^41,42^, and can also bind to chromatin in response to hormones and biotic and abiotic stress conditions^43,44^; AGO5 interacts with miR156 to control flowering time^45^; and AGO10 regulates the development of shoot apex meristem^46^. Additionally, AtAGO1, 2, 3, and 7 play an important role in plant adaptation to salt stress^46^, and AtAGO7 plays a critical role in the transition from the juvenile to the adult stage during plant growth^47^. In addition, AGO1, AGO2, AGO3, AGO4, and AGO5 in Arabidopsis are involved in the antiviral defence response^48,49^.

Structural visualization can provide an understanding of the differences between CqAGO and AtAGO. As demonstrated by the secondary and 3D structures, AGO generally has several important domains: N domain, L2, PAZ, MID, and PIWI. The 3D model of the AGO protein shows a bilobal protein, wherein the N domain, the linker region, and the PAZ domain form the N-terminal lobe, while the MID and PIWI domains constitute the C-terminal lobe^50^, with a cleft between the two. The central cleft is composed of positively charged aa residues that can promote the binding of negatively charged small RNAs^51^. The N domain of AGO is involved in the cleavage of target RNA and the dissociation of sRNA double strands^9,52^. Further, L2 can connect the PAZ domain with MID, N domain with DUF1785 domains to help stabilize the entire protein structure^51^. The MID domain has a binding region named nucleotide-specific loop, which can recognize and bind the 5′ nucleotide of sRNA, making the binding of AGO and sRNA highly specific. For example, AtAGO1 containing the MID region preferentially recognizes the sRNA with a 5′ U, whereas AtAGO4 and AtAGO5 preferentially recognize sRNAs with 5′ A and 5′ C, respectively^36^. Furthermore, the QF-V motif of PIWI domain in AtAGO1 and AtAGO2 helps to recognize the #15 base pair in the sRNA duplex, and is essential for the effective sorting of miRNA into AtAGO1 and AtAGO2^53^. In this study, all CqAGO members in the AtAGO1/5/10 clade and CqAGO4c, 6a and 6b in the AGO4/6/8/9 clade contained the MID domain, therefore, it can be inferred that they may recognize sRNAs through the QF-V motif or the MID domain. However, all CqAGOs grouped in the AtAGO2/3/7 clade did not contain an identifiable MID domain, suggesting that they may recognize sRNA through the QF-V motif only.

Furthermore, all CqAGOs contain PAZ and PIWI domains. The PAZ and PIWI domains are important domains that form RISC; the PAZ domain can recognize the 2-nt 3′ end of sRNA^54^, while the PIWI domain can bind the 5′ end of siRNA to the target RNA, cleaving the target RNA complementary to the sRNA sequence^55,56^. The DEDD/H in the PIWI domain is required for RNase H-like endonuclease activity^57^. Most CqAGOs also exhibit the DEDD/H catalytic tetrad except for CqAGO2/3, in which the first D was replaced by N, and CqAGO5e, which is a short protein that lacks the last catalytic residue D/H. Incomplete catalytic residues may fail CqAGO2/3 and 5e to perform slice activity, may induce gene silencing by other means, or help in performing novel functions. However, studies have shown that even if the AGO protein has a conserved catalytic tetrad, it may not necessarily have endonuclease activity. It has been determined that AtAGO1, AtAGO2, AtAGO4, AtAGO7, and AtAGO10 have endonuclease activity^57^; however, other AGOs do not display endonuclease activity and may regulate PTGS by inhibiting the translation of target RNA^58^. In addition, the H798 residue in the PIWI domain is important for cleavage function, and its lack thereof leads to cleavage deficiency in AtAGO1^59^. The P residue replaces the H residue in barley, adding HvAGO5b to act as a chromatin modifier^38^. In the AGO4/6/8/9 clade, CqAGO4b, 4c, 4d, 4e, 4f, 6a, 6b, and 8/9 contain P residues (Fig. 4A), which suggests that these CqAGOs lack cleavage function and may act as chromatin modifiers. The N residues in AtAGO1 and OsAGO1 in the PIWI domain may preferentially bind to 5′ U 21-nt sRNA^36,60^, whereas in the CqAGO family, 11 of 21 CqAGOs have retained the N residues, including 1a, 1b, 4a, 4b, 4c, 4d, 4e, 4f, 8/9, 10a, and 10b (Fig. 4B). This indicates that these AGOs may have similar preferences. AGOs in the same clade of quinoa and Arabidopsis were conserved in terms of aa sequence, secondary, and 3D structures, suggesting greater functional similarity among them. Nonetheless, these results warrant further investigation.

As a member of the ribonuclease III enzyme family, DCL can regulate gene expression and participate in antiviral defence via the RNAi pathway^37^. Arabidopsis encodes four DCL proteins that produce different sRNAs. AtDCL1 is related to the production of miRNAs, which can regulate gene expression in fundamental biological processes, such as development and metabolism^11,13^. In contrast, AtDCL2, AtDCL3, and AtDCL4 process long dsRNA into 22-, 24- and 21-nt-long siRNA, respectively^61^. Furthermore, AtDCL2 and AtDCL4 are also involved in antiviral defence response^62^, and AtDCL3 mainly guides chromatin modification and maintains genome stability^63^. DCL proteins mainly include six domains: DEXDc, HELICc, Dicer-dimer, PAZ, RIBOc, and DSRM. PAZ and RIBOc are essential for the removal of siRNA from the end of the dsRNA molecule^64,65^. In the AtDCL4 model, the spatial arrangement of PAZ and RIBOc helps ‘measure’ cleaved dsRNA^37^. Studies have shown that catalytic residues N and K of RNase III A and RNase III B in RIBOc are highly conserved^66^. In the AtDCL4–dsRNA complex, N and K residues can interact with dsRNA^37^, while in the yeast RNase III—RNA complex, the N and K residues can interact with the 5′-phosphate group of the cleavage bond^67^. The N and K of RNase III A and RNase III B in CqDCL are highly conserved (Fig. 5B,C), indicating that N and K in CqDCL may also be involved in the cleavage of the phosphodiester bond. Consistent with the prediction of AtDCL2 in the same clade^68^, only one DSRM domain was predicted for CqDCL2a and CqDCL2b. In terms of structure, they did not contain a second DSRM domain. DSRM may be involved in protein–protein interactions, such as the specific binding of AtDCL to the HYPONASTIC LEAVES (HYL) protein family^69^. As the DSRM domain also mediates the transfer of sRNA to the appropriate AGO protein^70^, the partial deletion of the DSRM domain may affect the binding of DCL and downstream genes of the RNAi pathway.

Single-stranded RNA molecules are used by RDR as templates to synthesize dsRNA, which is then cleaved by DCL into secondary siRNA to enhance and maintain the silent state of the target RNA^71^. Studies have shown that RDR can regulate reproductive development in Arabidopsis, including female gametophyte development, maternal-to-zygotic transition, self-fertilization, and double fertilization^72–75^. Furthermore, RDR is involved in the antiviral response. Thus, AtRDR1, AtRDR2, and AtRDR6 have lost or altered functions, thereby increasing susceptibility to a variety of plant viruses and viral RNA accumulation^76^. Under various stress conditions, the *AtRDR6* gene is the most sensitive, it is induced in response to high temperatures and repressed during long exposure to salt or cold stress, while *AtRDR1* and *AtRDR5* expression decrease during prolonged exposure to high salinity or low temperatures^77^.

The RDR protein has only one conserved catalytic domain: RdRP. Of the three main types of RDRs (RDRα, RDRβ, and RDRγ), plants only contain RDRα and RDRγ^78^. In Arabidopsis, AtRDR1, 2, and 6 belong to RDRα and contain the typical C-terminal catalytic motif, DLDGD. RDR1, RDR2, or RDR6 can mediate the production of a variety of viral siRNAs and play an important role in defence against viruses in plants^79–83^. In quinoa, CqRDR1, 2a, 2b, 6a, and 6b, belonging to the AtRDR1/2/6 clade, share the DLDGD motif^84^. This similarity in structure implies that CqRDR1, 2a, 2b, 6a, and 6b play a role in plant defence responses against pathogens. Owing to their high sequence similarity, AtRDR3, 4, and 5, also named RDR3a, 3b, and 3c, belong to RDRγ^78^ and share an uncharacteristic catalytic DFDGD motif^84^. The CqRDR motif belonging to the AtRDR3 clade is DL/YDGD, in which F is replaced by L/Y, with an unknown function. Each CqRDR has an extension, except for AtRDR1, which may indicate the diversification of the quinoa RDR family.

Subcellular localization is important to understand the molecular functions of AGO, DCL, and RDR. Furthermore, miRNAs (such as miR-29b) that bind to AGO, contain a nuclear localization signal (NLS)^85^. AGO participates in the formation of heterochromatin by recruiting methyltransferase and acetyltransferase onto chromatin to perform TGS in various organisms and participates in transcriptional silencing in the nucleus^86^. Similarly, most CqAGOs are localized to the nucleus (Table S9). Studies have shown that DCL1-GFP and DCL4-GFP fusion proteins are localized in the nucleus^69^. Non-classical NLS have detected in the dsRNA C-terminal binding domains of DCL1 and DCL4^86^, and these NLSs can likely guide DCL1 and DCL4 to the nucleus. This study predicted that all CqDCL members are localized in the nucleus, further implying that CqAGOs and CqDCLs may participate in the RNAi pathway.

In this study, the tissue-specific and abiotic stress expression patterns of *CqAGO*, *CqDCL* and *CqRDR* genes were investigated. RNA-seq results showed that most of these genes were expressed in five tissues including dry seeds, seedlings, internode stems, inflorescences and leaves, but the expression of the same gene varied in different tissues, indicating that these genes may be involved in different developmental processes. Studies have shown that *AtAGO1* regulates leaf development, and *AtAGO1* together with *AtAGO10* regulates floral stem cell termination through miR172 and miR165/166^87^. *CqAGO1a* and *CqAGO1b* (the homologous genes of *AtAGO1*) were highly expressed in five tissues, thus, we speculated that they may be involved in the development or maintenance of leaves and flowers. *OsDCLs* and *AtDCLs* were expressed in different tissues, *AtDCL1*, *AtDCL3*, and *AtDCL4* were expressed at higher levels in flowers^88^. Similarly, *CqDCL1a, CqDCL1b, and CqDCL4a* were also expressed in all five tested tissues, and showed the highest expression in inflorescences. *OsRDR6* is required for floral organ development^89^, and *CqRDR6a* and *CqRDR6b* are also highly expressed in inflorescences in the RNA-seq results, suggesting that *CqDCLs* and *CqRDRs* are involved in floral organ development in quinoa. Plants resist the effects of stress in a variety of ways. In response to stresses such as drought, salt, and heat, the expression of many plant genes changes. For example, the expression of *OsDCL* was slightly inhibited under drought, cold and salt stress^88^. Under salt stress, the expression of *AtDCL1* in roots and shoots showed a downward trend, and the expression of *AtDCL4* decreased with the prolongation of salt treatment time^88^. Similar to these results, based on the result of the RNA-seq, the expression levels of most *CqDCLs* in both tissues were decreased in all stresses, which suggested that most *CqDCLs* are involved in abiotic stress responses.

## Conclusion

In this study, 21 *CqAGO*, eight *CqDCL* and 11 *CqRDR* genes were identified in *C. quinoa*. Based on bioinformatics analyses, we aimed to improve the understanding of these gene families, including their genomic location, phylogenetic relationship, domain components, 3D structure, related functional annotations, subcellular localization and expression patterns. We show that these gene families have the potential to regulate gene transcription and translation, which may indicate a role in the typical RNAi pathway in quinoa. This is the first report that provides insight into important gene families involved in the biogenesis of sRNA in quinoa, which paves the way for further functional characterization of these genes. This information can be used to improve stress resistance and yield quality in quinoa. However, in addition to our bioinformatics analyses, further investigation is needed to confirm the functions of these proteins and pinpoint their roles in the involvement of the RNAi pathway in growth and development and disease resistance in quinoa.

## Methods

### Sequence acquisition and database search

Sequence information of the *AGO*, *DCL*, and *RDR* genes in Arabidopsis was obtained from TAIR (http://www.arabidopsis.org) (Table S1). The coding sequences (CDS), protein sequences, CDS length, and peptide length in quinoa corresponding to the primary transcripts of Arabidopsis homologous genes were downloaded from the Plant Comparative Genomics portal Phyzome 13 *Chenopodium quinoa* v1.0 database (https://phytozome-next.jgi.doe.gov/)^90^. The description and chromosomal location of *CqAGO*-, *CqDCL*-, and *CqRDR*-related sequences were determined using Expasy (https://www.expasy.org/), the chromosome location of these genes were represented using online tool Mapgene2chrom (http://mg2c.iask.in/mg2c_v2.1/)^91^.

### Phylogenetic and structural analyses, gene ontology annotation, and subcellular localization

Phylogeny analysis was performed using the Phylogeny.fr web server (http://phylogeny.lirmm.fr/phylo_cgi/index.cgi)^92^, the *CqAGO*, *CqDCL*, and *CqRDR* genes predicted in the quinoa genome were named according to their phylogenetic relationship with the members of the same protein family in Arabidopsis. Multiple sequence alignment (MSA) was performed using Clustal Omega (https://www.ebi.ac.uk/Tools/msa/clustalo/)^93^, and multiple alignment viewer (Mview) (https://www.ebi.ac.uk/Tools/msa/mview/)^93^ was used to visualize the conserved residues and domains of related proteins. To identify the similarity of two sequences, we used the pairwise sequence alignment tool EMBOSS Needle (https://www.ebi.ac.uk/Tools/psa/)^93^. The simple modular architecture research tool (SMART) (http://smart.embl-heidelberg.de/)^94^ in normal SMART mode was used for the analysis of protein domains, and illustrator for biological sequences (IBS) (http://ibs.biocuckoo.org/)^95^ were used for domain visualization. The gene intron structures were predicted using the online software gene structure display server (GSDS) v.2.0 (http://gsds.gao-lab.org/)^96^. GO annotation was performed using Expasy (https://www.expasy.org/), and protein localization was predicted using the plant subcellular localization integrative predictor (PSI) (http://bis.zju.edu.cn/psi/).

### 3D structure modeling and verification

SWISS-MODEL, a homology-based modeling software (https://beta.Swissmodel.Expasy.Org/)^97^, was used to predict the 3D structure of proteins, and the template was checked using SAVES v 6.0 (https://saves.mbi.ucla.edu/). The Python molecular graphics system (PyMOL) (https://pymol.org/2) was used to visualize protein structures.

### Expression profile analysis of *CqAGOs*, *CqDCLs* and *CqRDRs*

Transcriptome data of quinoa-related tissues were downloaded from NCBI’s SRA database, including different tissues (SRP116149), and different stresses of drought, heat, salt, and low phosphorus in the root and shoot (SRS1538629). The R package pheatmap was used to cluster and visualize the data with the following parameter settings: distance measure, Euclidean; clustering method, Median. Kallisto was used to calculate the expression level. Tissue expression heatmaps were drawn using TBtools^98^.

### Plant materials, RNA extraction, PCR amplification and electrophoresis

Quinoa (QQ74) was provided by the Agricultural College, Shanxi Agricultural University, grown in growth chambers at 24 °C day/22 °C night, 16 day length. Dry seeds, one-week-old seedlings, stems, leaves, and inflorescences from six-week-old plants were collected and frozen in liquid nitrogen. Total RNA from different tissues was extracted according to the instructions of Trizol, and cDNA was obtained by reverse transcription. The genes that the reads of RNA-sequencing (RNA-seq) were greater than 100 in all five tissues were selected, designed primers, and amplified. After PCR amplification, PCR products were analysed via electrophoresis on 1.5% agarose gels, and the amplified target fragment was observed with Quantity One software. Primers were listed in Table S10. The complete electrophoretic diagrams were shown in Fig. S3. Experimental research on the plant(s)/plant material complied with the relevant institutional, national, and international guidelines and legislation.

## Supplementary Information

Supplementary Information.

## Data Availability

All data generated or analysed during this study are included in this published article and its supplementary information files.
