# Supplementary material for: Genome-wide identification, characterization and expression analysis of AGO, DCL, and RDR families in Chenopodium quinoa
Source: Sci Rep. 2023 Mar 4;13:3647. doi: 10.1038/s41598-023-30827-1 (PMC9985633; doi:10.1038/s41598-023-30827-1)
Supplement: Supplementary file 1 — Supplementary Information. [file 41598_2023_30827_MOESM1_ESM.pdf]

Supplementary Material

Figure S1. Chromosome location of *CqAGO* (red), *CqDCL* (black), and *CqRDR* (green) genes. The chromosome number is shown at the top of each bar, and the chromosome length indicating scale is shown on the left.

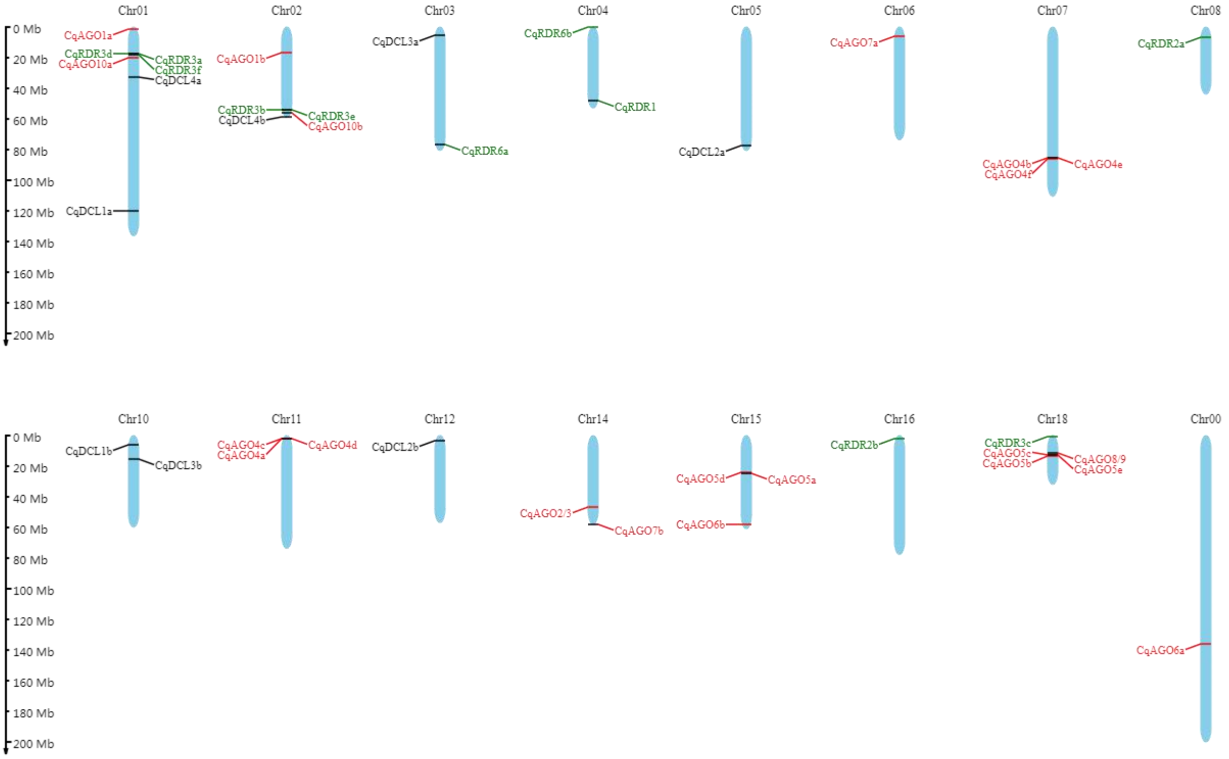

Figure S2. Three-dimensional structure prediction of AtAGO4/6/8/9 clade, as modeled by SWISS-MODEL. PAZ (yellow), Mid (red) and PIWI (blue) domains as predicted by SMART and Pfam displayed. DEDD/H is marked by magenta spheres.

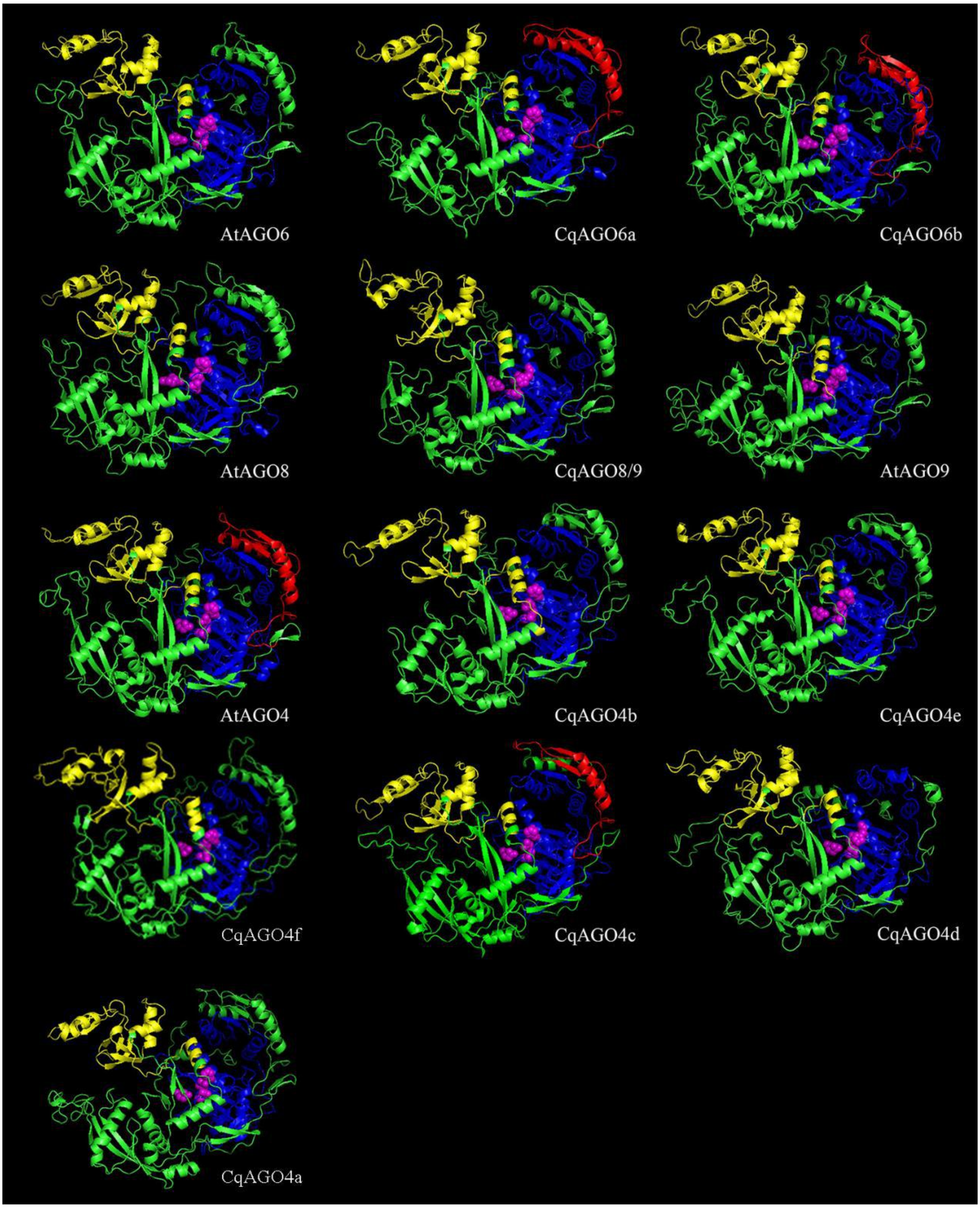

Figure S3. Electrophoresis Analysis of *CqAGOs*, *CqDCLs*, and *CqRDR* expression in different normal quinoa tissues (From left to right were dry seeds, internode stems, inflorescences, leaves, and seedlings). The marker contained ten bands with sizes of 10000, 5000, 3000, 2000, 1500, 1000, 750, 500, 250, and 100 bp. Markers in all electrophoresis diagrams were the same.

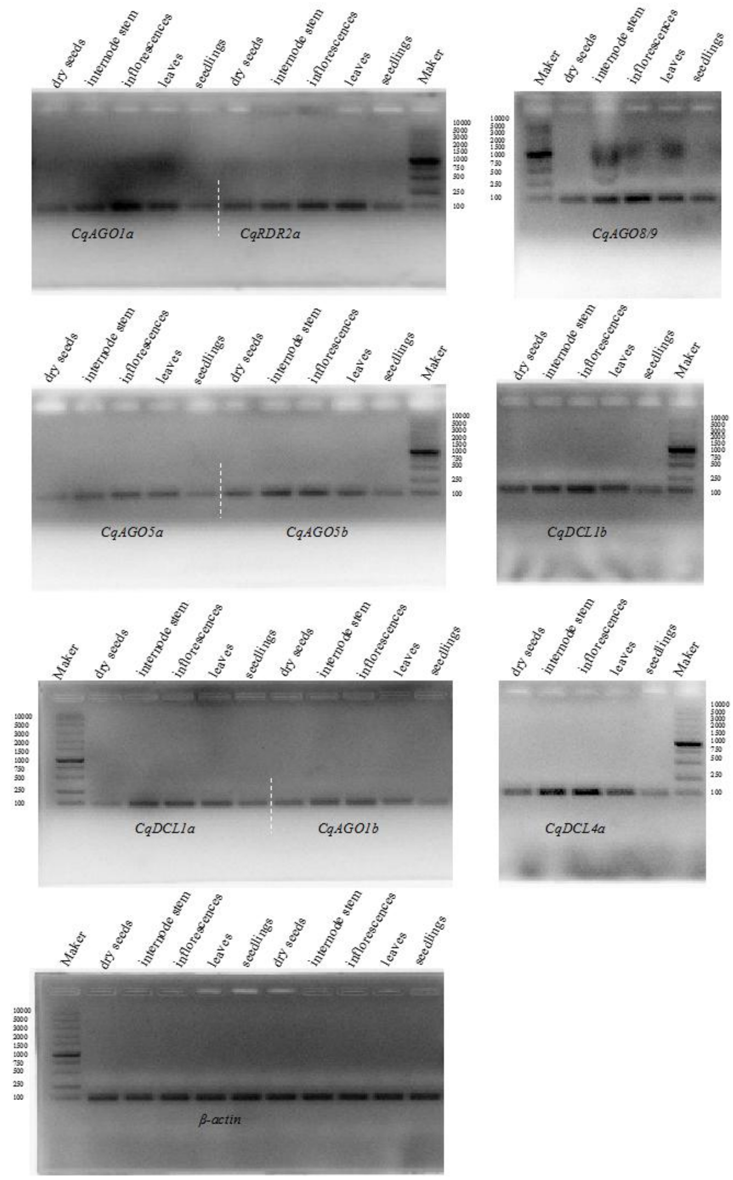

Table S1. Accession numbers of *AtAGO*, *AtDCL*, and *AtRDR* gene IDs.

**Assigned name of genes**	**Gene ID**
AtAGO1	AT1G48410.2
AtAGO2	AT1G31280.1
AtAGO3	AT1G31290.1
AtAGO4	AT2G27040.1
AtAGO5	AT2G27880.1
AtAGO6	AT2G32940.1
AtAGO7	AT1G69440.1
AtAGO8	AT5G21030.1

AtAGO9	AT5G21150.1
AtAGO10	AT5G43810.1
AtDCL1	AT1G01040.1
AtDCL2	AT3G03300.1
AtDCL3	AT3G43920.2
AtDCL4	AT5G20320.1
AtRDR1	AT1G14790.1
AtRDR2	AT4G11130.1
AtRDR3a	AT2G19910.1
AtRDR3b	AT2G19920.1
AtRDR3c	AT2G19930.1
AtRDR6	AT3G49500.1

Table S2. Domain structure of CqAGO proteins, as identified by domain search by SMART, with Pfam overlaps of higher confidence.

Protein	N domain	DUF1785	PAZ	L2	MID	PIWI
CqAGO1a	181 - 316	325 - 377	382 - 522	526 - 572	582 - 661	679 - 1000
	(3.4e-35)	(6.96e-25)	(0.00155)	(1.1e-15)	(1.9e-07)	(8.65e-129)
CqAGO1b	147 - 282	291 - 330	358 - 483	487 - 533	543 - 622	640 - 961
	(3.2e-35)	(0.000472)	(0.00588)	(1e-15)	(1.8e-7)	(6.66e-130)
CqAGO2/3		3 - 39	44 - 192			351 - 624
		(4.57)	(0.357)			(1.85e-73)
CqAGO4a	27 - 188		237 - 365	374 - 420		527 - 813
	(1.6e-23)		(2.4e-17)	(5.6e-09)		(1.52e-92)
CqAGO4b	23 - 149	159 - 210	214 - 341	341 - 388		473 - 779
	(7.2e-18)	(2.2e-13)	(2.9e-09)	(1.3e-10)		(8.42e-109)
CqAGO4c	23 - 184	194 - 245	249 - 381	389 - 436	442 - 509	533 - 839
	(2.4e-28)	(2.1e-10)	(1.1e-21)	(1.5e-10)	(2.7e-7)	(8.97e-104)
CqAGO4d	14 - 177	183 - 233	237 - 369	377 - 422		449 - 736
	(3.1e-27)	(1.69e-14)	(1e-19)	(3.1e-07)		(2.87e-93)
CqAGO4e	23 - 181	191 - 243	247 - 379	387 - 434		520 - 826
	(2.1e-27)	(9.71e-15)	(7.3e-20)	(2.1e-09)		(2.74e-99)
CqAGO4f	23 - 165	175 - 227	231 - 363	371 - 418		516 - 787
	(2e-25)	(1.24e-12)	(2.1e-20)	(1.9e-09)		(2.14e-78)
CqAGO5a	29 - 164	174 - 234	239 - 366	372 - 418	428 - 505	514 - 825
	(2.2e-31)	(1.2e-14)	(0.00466)	(1.8e-14)	(7.1e-09)	(2.84e-115)
CqAGO5b	49 - 184		237 - 362	368 - 414	424 - 502	511 - 828
	(3.9e-32)		(0.00428)	(4.2e-14)	(2.3e-10)	(2.83e-124)

CqAGO5c	107 - 241 (3.5e-26)	250 - 301 (4.03e-21)	314 - 436 (0.00000996)	442 - 488 (3.9e-15)	495 - 571 (4e-11)	583 - 893 (4.87e-114)
CqAGO5d	111 - 243 (4.2e-32)	253 - 304 (4.70e-22)	311 - 438 (0.0253)	444 - 491 (5.6e-14)	501 - 580 (2.1e-10)	583 - 897 (4.23e-123)
CqAGO5e	76 - 184 (2.2e-10)		207 - 337 (0.0341)	343 - 390 (3.4e-14)	400 - 479 (6.9e-9)	482 - 750 (1.27e-30)
CqAGO6a		154 - 204 (9.22e-7)	208 - 339 (6.9e-23)	348 - 394 (7.6e-14)	401 - 484 (1.2e-07)	491 - 805 (3.12e-103)
CqAGO6b	11 - 162 (2.6e-18)	172 - 224 (3.41e-10)	228 - 359 (1.5e-22)	368 - 414 (7.3e-13)	421 - 504 (1.3e-07)	511 - 861 (1.82e-95)
CqAGO7a	95 - 241 (2.1e-24)	250 - 302 (4.36e-17)	310 - 444 (0.000105)			598 - 906 (1.18e-103)
CqAGO7b	96 - 242 (2.1e-25)	251 - 303 (7.08e-17)	311 - 445 (0.0000107)			599 - 907 (1.59e-104)
CqAGO8/9	82 - 170 (8.2e-13)	180 - 232 (1.02e-17)	236 - 366 (9e-26)	375 - 421 (9.1e-12)		518 - 826 (1.38e-113)
CqAGO10a	97 - 231 (5.7e-28)	240 - 292 (1.36e-18)	297 - 429 (0.0334)	433 - 479 (1.6e-15)	489 - 569 (3.9e-12)	581 - 902 (5.58e-123)
CqAGO10b	97 - 231 (7.7e-28)	240 - 292 (1.36e-18)	297 - 429 (0.0334)	433 - 479 (1.6e-15)	489 - 569 (3.9e-12)	581 - 902 (1.2e-122)

Table S3. Domain structure of CqDCL proteins, as identified by domain search by SMART, with Pfam overlaps of higher confidence.

Protein	DEXDc	HELICc	Dicer_dimer	PAZ	RIBOc	RIBOc	DSRM	DSRM
CqDCL1a	234 - 437 (8.3e-19)	676 - 762 (3.88e-10)	835 - 926 (7.9e-22)	1183 - 1340 (3.07e-29)	1355 - 1539 (1.88e-37)	1575 - 1710 (1.6e-40)	1714 - 1775 (1.12e-8)	1801 - 1874 (3e-9)
CqDCL1b	222 - 425 (8.3e-19)	662 - 748 (5.65e-12)	821 - 912 (6e-22)	1169 - 1326 (3.07e-29)	1341 - 1525 (3.98e-36)	1561 - 1717 (1.19e-48)	1721 - 1782 (1.12e-8)	1808 - 1881 (2.31e-10)
CqDCL2a	18 - 216 (5.87e-19)	358 - 445 (7.18e-14)	513 - 595 (8.7e-16)	786 - 924 (9.43e-7)	945 - 1098 (8.82e-20)	1131 - 1286 (8.01e-41)	1290 - 1354 (0.000638)	
CqDCL2b	18 - 216 (2.74e-19)	327 - 423 (3.38e-10)	491 - 573 (4.4e-15)	762 - 900 (0.00000217)	921 - 1074 (9.31e-19)	1107 - 1262 (1.30e-40)	1266 - 1329 (0.0131)	
CqDCL3a				678 - 866 (0.00712)	883 - 1056 (1.19e-25)	1092 - 1244 (4.01e-32)		
CqDCL3b				191 - 379 (0.0143)	396 - 569 (3.61e-25)	605 - 757 (4.01e-32)		
CqDCL4a	30 - 239 (1.72e-25)	407 - 495 (1.87e-12)	559 - 646 (5.9e-23)	831 - 974 (0.00247)	995 - 1163 (4.51e-26)	1199 - 1350 (9.44e-29)	1354 - 1418 (0.00314)	1552 - 1626 (2.27e-9)
CqDCL4b	30 - 239 (2.76e-24)	406 - 494 (1.62e-12)	558 - 645 (7.7e-26)	830 - 973 (0.00438)	994 - 1162 (4.51e-26)	1198 - 1349 (2.14e-27)	1353 - 1417 (0.00429)	1552 - 1626 (2.27e-9)

Table S4. Domain structure of CqRDR proteins, as identified by domain search by SMART, with Pfam overlaps of higher confidence.

Protein	RRM	RdRP
CqRDR1	4-82(0.0328)	369-940 (3.9e-187)
CqRDR2a	12-90(0.0111)	355-912 (1.2e-178)
CqRDR2b		8-581 (2.7e-182)
CqRDR3a		224-728 (5.1e-93)
CqRDR3b		229-868 (2.4e-113)
CqRDR3c		2-474 (2.1e-68)
CqRDR3d		216-720 (4e-70)
CqRDR3e		280-782 (2e-78)
CqRDR3f		185-811 (7.4e-108)
CqRDR6a		419-983 (1.1e-169)
CqRDR6b		419-1004 (1.2e-186)

Table S5. Validation of predicted 3D structures of AtAGO and CqAGO proteins. Validation by GMQE, QMEAN, PROCHECK, ERRAT, Verify 3D, and WHATCHECK. The second column indicates the template used for the model and coverage.

Protein	Template used (coverage)	QMEAN	GMQE	Seq Identity	Seq Similarity	Residues in favored region (PROCHECK)	Overall quality factor (ERRAT)	The residues have averaged 3D-1D score >= 0.2 (Verify 3D)	Ramachandran z-score (WHATCHECK)
AtAGO1	Protein argonaute-1-4kre.1.A (0.79)	-1.87	0.55	43.6%	0.41	89.2%	90.4988	94.29%	-1.380
AtAGO2	Protein argonaute-1-4z4h.1.A (0.81)	-2.59	0.52	31.11%	0.36	89.8%	88.3777	86.92%	-0.987
AtAGO3	Protein argonaute-2-5js2.1.A (0.69)	-2.42	0.43	31.52%	0.36	90.9%	87.4556	92.13%	-1.469
AtAGO4	Protein argonaute-2-5js2.1.A (0.87)	-1.86	0.60	33.58%	0.37	88.4%	88.4248	95.64%	-1.337
AtAGO5	Protein argonaute-4-6oon.1.A (0.83)	-2.27	0.56	42.12%	0.40	89.1%	92.8136	92.39%	-1.662
AtAGO6	Protein argonaute-1-4kre.1.A (0.91)	-1.95	0.62	32.71%	0.36	89.0%	86.3636	87.41%	-1.506
AtAGO7	Protein argonaute-2-4ola.1.A (0.82)	-2.37	0.54	37.5%	0.39	88.0%	86.6989	83.99%	-1.679
AtAGO8	Protein argonaute-2-4f3t.1.A (0.92)	-2.66	0.61	31.15%	0.36	87.7%	86.0349	85.04%	-1.931

AtAGO9	Protein argonaute-1-4kxt.1.A (0.92)	-2.60	0.62	31.88%	0.36	85.6%	88.6499	95.24%	-1.583
AtAGO10	Protein argonaute-2-4ola.1.A (0.84)	-2.09	0.59	43.29%	0.41	89.6%	90.4306	94.44%	-1.202
CqAGO1a	Protein argonaute-2-4z4h.1.A (0.79)	-2.39	0.53	42.38%	0.41	88.9%	91.8224	92.00%	-1.523
CqAGO1b	Protein argonaute-1-5w6v.1.A (0.81)	-2.82	0.55	42.49%	0.41	89.4%	90.6176	91.61%	-1.172
CqAGO2/3	Protein argonaute-1-4kre.1.A (0.96)	-2.77	0.64	31.62%	0.36	89.1%	87.5614	81.02%	-1.361
CqAGO4a	Protein argonaute-2-5t7b.1.B (0.91)	-3.13	0.60	29.87%	0.35	88.7%	88.6108	91.19%	-1.770
CqAGO4b	Protein argonaute-2-5js2.1.A (0.95)	-2.28	0.64	30.90%	0.36	88.9%	86.8895	87.27%	-1.795
CqAGO4c	Protein argonaute-2-5js2.1.A (0.88)	-2.40	0.62	32.12%	0.37	89.6%	88.404	90.25%	-1.734
CqAGO4d	Protein argonaute-2-4z4h.1.A (0.94)	-3.03	0.63	31.50%	0.36	87.8%	85.8289	88.24%	-1.954
CqAGO4e	Protein argonaute-2-5js2.1.A (0.93)	-2.63	0.64	31.13%	0.36	88.7%	87.0617	89.95%	-1.597
CqAGO4f	Protein argonaute-2-6mdz.1.A (0.95)	-3.22	0.64	29.30%	0.35	87.3%	88.491	86.80%	-2.025
CqAGO5a	Protein argonaute-1-4kxt.1.A (0.94)	-2.92	0.68	41.40%	0.40	88.0%	88.1563	94.71%	-1.349
CqAGO5b	Protein argonaute-2-w5q.1.A (0.92)	-2.68	0.66	39.98%	0.40	85.8%	86.0149	90.18%	-1.237
CqAGO5c	Protein argonaute-1-4kre.1.A (0.88)	-2.01	0.61	38.66%	0.39	88.8%	88.5817	92.76%	-1.207
CqAGO5d	Protein argonaute-2-5js2.1.A (0.87)	-1.46	0.63	41.39%	0.41	89.1%	85.8911	89.77%	-1.537
CqAGO5e	Protein argonaute-2-4z4h.1.A (0.89)	-2.96	0.57	36.12%	0.38	88.6%	91.5361	83.24%	-1.573
CqAGO6a	Protein argonaute-2-4ola.1.A (0.93)	-2.69	0.62	31.97%	0.36	86.0%	87.3434	86.73%	-1.899
CqAGO6b	Protein argonaute-2-4f3t.1.A (0.89)	-3.45	0.60	31.30%	0.36	88.7%	86.7052	85.67%	-1.494
CqAGO7a	Protein argonaute-2-5js2.1.A (0.87)	-2.31	0.60	37.14%	0.38	88.0%	89.2382	92.38%	-1.844
CqAGO7b	Protein argonaute-2-5js2.1.A (0.87)	-2.44	0.60	36.89%	0.38	89.5%	88.7694	88.34%	-1.644
CqAGO8/9	Protein argonaute-2-4w5q.1.A (0.81)	-2.73	0.52	31.56%	0.36	89.3%	89.7078	89.40%	-1.390
CqAGO10a	Protein argonaute-2-4f3t.1.A (0.88)	-2.25	0.63	42.79%	0.41	89.9%	89.2052	93.58%	-1.328
CqAGO10b	Protein argonaute-2-4f3t.1.A (0.88)	-2.34	0.63	42.67%	0.41	89.3%	90.8434	90.67%	-1.668

Table S6. Gene ontology annotation of the *CqAGO* genes.

Genes	GO term		
CqAGO1a	GO:0005737	[CELLULAR COMPONENT]	cytoplasm
	GO:0016070	[BIOLOGICAL PROCESS]	RNA metabolic process
	GO:0035195	[BIOLOGICAL PROCESS]	gene silencing by miRNA
	GO:0035198	[molecular_function]	miRNA binding
CqAGO4b	GO:0003676	[molecular_function]	nucleic acid binding
	GO:0004523	[molecular_function]	RNA-DNA hybrid ribonuclease activity
CqAGO5a	GO:0000956	[BIOLOGICAL PROCESS]	nuclear-transcribed mRNA catabolic process
	GO:0010501	[BIOLOGICAL PROCESS]	RNA secondary structure unwinding
	GO:0022603	[BIOLOGICAL PROCESS]	regulation of anatomical structure morphogenesis
	GO:0031054	[BIOLOGICAL PROCESS]	pre-miRNA processing
	GO:0035278	[BIOLOGICAL PROCESS]	miRNA mediated inhibition of translation
	GO:0035280	[BIOLOGICAL PROCESS]	miRNA loading onto RISC involved in gene silencing by miRNA
	GO:0045944	[BIOLOGICAL PROCESS]	positive regulation of transcription by RNA polymerase II
	GO:1901342	[BIOLOGICAL PROCESS]	regulation of vasculature development
	GO:0001046	[molecular_function]	core promoter sequence-specific DNA binding
	GO:0003725	[molecular_function]	double-stranded RNA binding
	GO:0003727	[molecular_function]	single-stranded RNA binding
	GO:0035198	[molecular_function]	miRNA binding
	GO:0070063	[molecular_function]	RNA polymerase binding
	GO:0005622	[CELLULAR COMPONENT]	intracellular
	GO:0005634	[CELLULAR COMPONENT]	nucleus
	GO:0005829	[CELLULAR COMPONENT]	cytosol
	GO:0005844	[CELLULAR COMPONENT]	polysome
	GO:0032991	[CELLULAR COMPONENT]	protein-containing complex
	GO:0043229	[CELLULAR COMPONENT]	intracellular organelle
	GO:1990904	[CELLULAR COMPONENT]	ribonucleoprotein complex
CqAGO5b	GO:0003676	[molecular_function]	nucleic acid binding
CqAGO5d	GO:0003676	[molecular_function]	nucleic acid binding
CqAGO7a	GO:0003676	[molecular_function]	nucleic acid binding
	GO:0016246	[BIOLOGICAL PROCESS]	RNA interference
CqAGO7b	GO:0003676	[molecular_function]	nucleic acid binding
CqAGO8/9	GO:0003676	[molecular_function]	nucleic acid binding
CqAGO10a	GO:0003676	[molecular_function]	nucleic acid binding
CqAGO10b	GO:0003676	[molecular_function]	nucleic acid binding

Table S7. Gene ontology annotation of the *CqDCL* genes.

Genes	GO term		
CqDCL1b	GO:0003677	[molecular_function]	DNA binding
	GO:0003723	[molecular_function]	RNA binding
	GO:0004525	[molecular_function]	ribonuclease III activity
	GO:0005524	[molecular_function]	ATP binding
	GO:0005634	[CELLULAR COMPONENT]	nucleus
	GO:0005737	[CELLULAR COMPONENT]	cytoplasm
	GO:0016075	[BIOLOGICAL PROCESS]	rRNA catabolic process
	GO:0030422	[BIOLOGICAL PROCESS]	production of siRNA involved in RNA interference
CqDCL2a	GO:0003677	[molecular_function]	DNA binding
	GO:0003723	[molecular_function]	RNA binding
	GO:0004525	[molecular_function]	ribonuclease III activity
	GO:0005524	[molecular_function]	ATP binding
	GO:0005634	[CELLULAR COMPONENT]	nucleus
	GO:0005737	[CELLULAR COMPONENT]	cytoplasm
	GO:1990904	[CELLULAR COMPONENT]	ribonucleoprotein complex
	GO:0030422	[BIOLOGICAL PROCESS]	production of siRNA involved in RNA interference
CqDCL2b	GO:0003677	[molecular_function]	DNA binding
	GO:0004525	[molecular_function]	ribonuclease III activity
	GO:0005524	[molecular_function]	ATP binding
	GO:0005634	[CELLULAR COMPONENT]	nucleus
	GO:0005737	[CELLULAR COMPONENT]	cytoplasm
	GO:0030422	[BIOLOGICAL PROCESS]	production of siRNA involved in RNA interference
CqDCL3b	GO:0004525	[molecular_function]	ribonuclease III activity
	GO:0006396	[BIOLOGICAL PROCESS]	RNA processing
CqDCL4a	GO:0003677	[molecular_function]	DNA binding
	GO:0004525	[molecular_function]	ribonuclease III activity
	GO:0005524	[molecular_function]	ATP binding
	GO:0006396	[BIOLOGICAL PROCESS]	RNA processing
CqDCL4b	GO:0003677	[molecular_function]	DNA binding
	GO:0003723	[molecular_function]	RNA binding
	GO:0004520	[molecular_function]	endodeoxyribonuclease activity
	GO:0004525	[molecular_function]	ribonuclease III activity
	GO:0005524	[molecular_function]	ATP binding
	GO:0005634	[CELLULAR COMPONENT]	nucleus
	GO:0005737	[CELLULAR COMPONENT]	cytoplasm
	GO:1990904	[CELLULAR COMPONENT]	ribonucleoprotein complex

	GO:0006308	[BIOLOGICAL PROCESS]	DNA catabolic process
	GO:0012501	[BIOLOGICAL PROCESS]	programmed cell death
	GO:0016043	[BIOLOGICAL PROCESS]	cellular component organization
	GO:0016075	[BIOLOGICAL PROCESS]	rRNA catabolic process
	GO:0030422	[BIOLOGICAL PROCESS]	production of siRNA involved in RNA interference
	GO:0090305	[BIOLOGICAL PROCESS]	nucleic acid phosphodiester bond hydrolysis

Table S8. Gene ontology annotation of the *CqRDR* genes.

Genes	GO term		
CqRDR1	GO:0001172	[BIOLOGICAL PROCESS]	transcription, RNA-templated
	GO:0031047	[BIOLOGICAL PROCESS]	gene silencing by RNA
	GO:0030422	[BIOLOGICAL PROCESS]	production of siRNA involved in RNA interference
	GO:0070919	[BIOLOGICAL PROCESS]	production of siRNA involved in chromatin silencing by small RNA
	GO:0003676	[molecular_function]	nucleic acid binding
	GO:0003723	[molecular_function]	RNA binding
	GO:0003968	[molecular_function]	RNA-directed 5'-3' RNA polymerase activity
	GO:0016740	[molecular_function]	transferase activity
	GO:0016779	[molecular_function]	nucleotidyltransferase activity
	GO:0005634	[CELLULAR COMPONENT]	nucleus
	GO:0030880	[CELLULAR COMPONENT]	RNA polymerase complex
CqRDR2a	GO:0005634	[CELLULAR COMPONENT]	nucleus
	GO:0030880	[CELLULAR COMPONENT]	RNA polymerase complex
	GO:0003676	[molecular_function]	nucleic acid binding
	GO:0016779	[molecular_function]	nucleotidyltransferase activity
	GO:0030422	[BIOLOGICAL PROCESS]	production of siRNA involved in RNA interference
	GO:0032774	[BIOLOGICAL PROCESS]	RNA biosynthetic process
	GO:0070919	[BIOLOGICAL PROCESS]	production of siRNA involved in chromatin silencing by small RNA
CqRDR2b	GO:0016779	[molecular_function]	nucleotidyltransferase activity
	GO:0032774	[BIOLOGICAL PROCESS]	RNA biosynthetic process
CqRDR3a	GO:0005634	[CELLULAR COMPONENT]	nucleus
	GO:0030880	[CELLULAR COMPONENT]	RNA polymerase complex
	GO:0016779	[molecular_function]	nucleotidyltransferase activity
	GO:0030422	[BIOLOGICAL PROCESS]	production of siRNA involved in RNA interference
	GO:0032774	[BIOLOGICAL PROCESS]	RNA biosynthetic process
	GO:0070919	[BIOLOGICAL PROCESS]	production of siRNA involved in chromatin silencing by small RNA
CqRDR3b	GO:0005634	[CELLULAR COMPONENT]	nucleus
	GO:0030880	[CELLULAR COMPONENT]	RNA polymerase complex

	GO:0016779	[molecular_function]	nucleotidyltransferase activity
	GO:0030422	[BIOLOGICAL PROCESS]	production of siRNA involved in RNA interference
	GO:0032774	[BIOLOGICAL PROCESS]	RNA biosynthetic process
	GO:0070919	[BIOLOGICAL PROCESS]	production of siRNA involved in chromatin silencing by small RNA
CqRDR3e	GO:0005634	[CELLULAR COMPONENT]	nucleus
	GO:0030880	[CELLULAR COMPONENT]	RNA polymerase complex
	GO:0016779	[molecular_function]	nucleotidyltransferase activity
	GO:0030422	[BIOLOGICAL PROCESS]	production of siRNA involved in RNA interference
	GO:0032774	[BIOLOGICAL PROCESS]	RNA biosynthetic process
	GO:0070919	[BIOLOGICAL PROCESS]	production of siRNA involved in chromatin silencing by small RNA
CqRDR3f	GO:0005634	[CELLULAR COMPONENT]	nucleus
	GO:0030880	[CELLULAR COMPONENT]	RNA polymerase complex
	GO:0016779	[molecular_function]	nucleotidyltransferase activity
	GO:0030422	[BIOLOGICAL PROCESS]	production of siRNA involved in RNA interference
	GO:0032774	[BIOLOGICAL PROCESS]	RNA biosynthetic process
	GO:0070919	[BIOLOGICAL PROCESS]	production of siRNA involved in chromatin silencing by small RNA
CqRDR6a	GO:0005634	[CELLULAR COMPONENT]	nucleus
	GO:0030880	[CELLULAR COMPONENT]	RNA polymerase complex
	GO:0003676	[molecular_function]	nucleic acid binding
	GO:0016779	[molecular_function]	nucleotidyltransferase activity
	GO:0030422	[BIOLOGICAL PROCESS]	production of siRNA involved in RNA interference
	GO:0032774	[BIOLOGICAL PROCESS]	RNA biosynthetic process
	GO:0070919	[BIOLOGICAL PROCESS]	production of siRNA involved in chromatin silencing by small RNA
CqRDR6b	GO:0005634	[CELLULAR COMPONENT]	nucleus
	GO:0030880	[CELLULAR COMPONENT]	RNA polymerase complex
	GO:0003676	[molecular_function]	nucleic acid binding
	GO:0016779	[molecular_function]	nucleotidyltransferase activity
	GO:0030422	[BIOLOGICAL PROCESS]	production of siRNA involved in RNA interference
	GO:0032774	[BIOLOGICAL PROCESS]	RNA biosynthetic process
	GO:0070919	[BIOLOGICAL PROCESS]	production of siRNA involved in chromatin silencing by small RNA

Table S9. Location of CqAGO, CqDCL, and CqRDR proteins as predicted by PSI.

Protein name	Predicted location	Score	P value	Protein name	Predicted location	Score	P value
CqAGO1a	Nucleus.	0.866	0	CqDCL1a	Cytosol.	0.277	7.9378e-06
					Nucleus.	0.675	0
CqAGO1b	Nucleus.	0.872	0	CqDCL1b	Cytosol.	0.290	6.2157e-19

					Nucleus.	0.682	0
CqAGO2/3	Nucleus.	0.527	8.9088e-172	CqDCL2a	Nucleus.	0.814	0
CqAGO4a	Cytosol.	0.425	0	CqDCL2b	Membrane.<br>Nucleus.	0.428<br>0.538	0<br>7.1493e-192
CqAGO4b	Nucleus.	0.830	0	CqDCL3a	Cytosol.<br>Nucleus.	0.276<br>0.695	4.1827e-05<br>0
CqAGO4c	Nucleus.	0.974	0	CqDCL3b	Nucleus.	0.832	0
CqAGO4d	Nucleus.	0.772	0	CqDCL4a	Nucleus.	0.771	0
CqAGO4e	Nucleus.	0.798	0	CqDCL4b	Cytosol.<br>Nucleus.	0.324<br>0.676	2.414e-86<br>0
CqAGO4f	Nucleus.	0.917	0				
CqAGO5a	Mitochondrion.<br>Chloroplast.	0.205<br>0.161	6.0247e-153<br>3.0207e-50	CqRDR1	Nucleus.	0.730	0
CqAGO5b	Nucleus.	0.434	1.3235e-33	CqRDR2a	Cytosol.	0.359	3.5207e-195
CqAGO5c	Nucleus.	0.720	0	CqRDR2b	Mitochondrion.<br>Nucleus.	0.111<br>0.413	9.355e-08<br>3.0813e-16
CqAGO5d	Nucleus.	0.826	0	CqRDR3a	Cytosol.<br>Chloroplast.	0.466<br>0.140	0<br>6.1658e-14
CqAGO5e	Nucleus.	0.740	0	CqRDR3b	Nucleus.	0.690	0
CqAGO6a	Cytosol.<br>Mitochondrion.	0.341<br>0.415	4.5949e-138<br>0	CqRDR3c	Cytosol.<br>Chloroplast.	0.359<br>0.385	3.5207e-195<br>0
CqAGO6b	Cytosol.<br>Mitochondrion.	0.344<br>0.433	1.3217e-146<br>0	CqRDR3d	Cytosol.	0.655	0
CqAGO7a	Nucleus.	0.760	0	CqRDR3e	Cytosol.	0.687	0
CqAGO7b	Chloroplast.<br>Nucleus.	0.353<br>0.473	0<br>2.6934e-82	CqRDR3f	Cytosol.<br>Chloroplast.	0.557<br>0.153	0<br>1.1668e-32
CqAGO8/9	Nucleus.	0.963	0	CqRDR6a	Cytosol.<br>Nucleus.	0.385<br>0.510	4.7373e-294<br>6.307e-141
CqAGO10a	Cytosol.<br>Nucleus.	0.329<br>0.630	1.0923e-990	CqRDR6b	Cytosol.<br>Chloroplast.	0.568<br>0.161	0<br>1.2427e-49
CqAGO10b	Cytosol.<br>Nucleus.	0.4430.518	0<br>9.9469e-155				

Table S10. List of PCR primers.

Gene name	Forward primer (5'-3')	Reverse primer (5'-3')
CqAGO1a	CCTTGCAACAGTCTGGTG	AGTCATCGCTTGAATAGG
CqAGO1b	GAGTGAAGGCAAGTGTTT	CATATTACCACGGTGTGTA
CqAGO5a	ATGCAGTGATTGGGAGAT	AATGCTTTGATAGTAACCC
CqAGO5b	AGCGGCGAGTGTTGATGG	ACACTTCGTTCCAACCCT
CqAGO8/9	TCCCAGTTCAATCAAGTC	TTCTTCTGAGCCACAATC
CqDCL1a	GAGGTTCAAAGTCCATT	CCTATCAACCTTACAACTC
CqDCL1b	CTTGGAGAAGCAGATACG	CTTGGAGAAGCAGATACG
CqDCL4a	ATCCTTCTACCGCCAAATC	GGCACCACCTTCCAGATA
CqRDR2a	ACCCTAGATGACAAACAGA	GTTTGAAGCCTCCAGTT
β-actin	GGTATTGGAACGGTGCCAGT	GGACTCGTGGTGCATCTCAA
